# Matrix metalloproteinase-10 promotes kidney fibrosis by transactivating β-catenin signaling

**DOI:** 10.1038/s41420-025-02521-w

**Published:** 2025-05-17

**Authors:** Xiaoli Sun, Qian Ren, Xi Liu, Huishi Tan, Zhanji Zhan, Enqing Lin, Yinyi Long, Xue Hong, Lili Zhou, Youhua Liu

**Affiliations:** 1 State Key Laboratory of Multi-organ Injury Prevention and Treatment, National Clinical Research Center of Kidney Disease, Division of Nephrology, Nanfang Hospital, Southern Medical University, Guangzhou, China; 2 Guangdong Provincial Key Laboratory of Renal Failure Research, Guangdong Provincial Institute of Nephrology, Guangzhou, China

**Keywords:** Mechanisms of disease, Cell signalling

## Abstract

Kidney fibrosis is characterized by excessive accumulation of extracellular matrix (ECM) and serves as a hallmark of chronic kidney disease (CKD). The turnover of ECM is controlled by a family of matrix metalloproteinases (MMPs), endopeptidases that play a crucial role in ECM remodeling and other cellular processes. In this study, we demonstrate that MMP-10 was upregulated in a variety of animal models of kidney fibrosis and human kidney biopsies from CKD patients. Bioinformatics analyses and experimental validation reveal that MMP-10 activated β-catenin in a Wnt-independent fashion. Knockdown of endogenous MMP-10 expression in vivo inhibited β-catenin activation and ameliorated kidney injury and fibrotic lesions, whereas over-expression of exogenous MMP-10 aggravated β-catenin activation and kidney fibrosis after injury. We found that MMP-10 cleaved and activated heparin-binding EGF-like growth factor (HB-EGF) via ectodomain shedding, leading to EGF receptor (EGFR) tyrosine phosphorylation and β-catenin transactivation via a cascade of events involving extracellular signal-regulated kinases and glycogen synthase kinase-3β. Consistently, treatment with erlotinib, a small-molecule EGFR inhibitor, effectively mitigated MMP-10-mediated kidney injury and fibrotic lesions in a dose-dependent fashion. Furthermore, β-catenin activation reciprocally upregulated the expression of MMP-10, thereby perpetuating kidney damage by forming a vicious cycle. Collectively, these results underscore that MMP-10 promotes kidney fibrosis through EGFR-mediated transactivating β-catenin in a Wnt-independent fashion. Our findings suggest that targeting MMP-10 could be a novel strategy for treatment of fibrotic CKD.

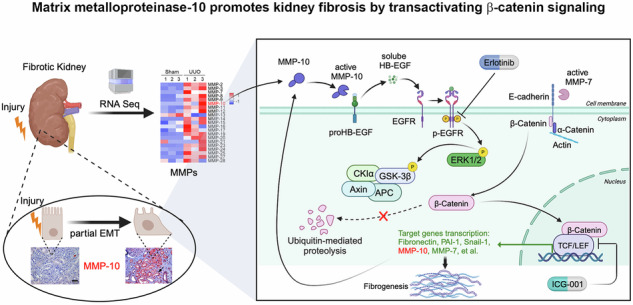

## Introduction

Chronic kidney disease (CKD) emerges as a global public health challenge, impacting about 850 million individuals worldwide [1, 2]. CKD is characterized by progressive kidney dysfunction and tissue fibrosis, and possesses a high risk of progressing to end-stage renal disease, a life-threatening condition requiring renal replacement therapy [3]. Despite regional and demographic disparities in morbidity and mortality, CKD becomes a prominent non-communicable contributor to global mortality [4, 5], and it is projected to be the fifth leading cause of death by 2040 [6]. The primary pathological features of CKD include renal inflammation, tubular atrophy, interstitial fibrosis, and vascular rarefaction [7, 8]. Among these, interstitial fibrosis represents the common pathological denominator determining disease progression across all CKD etiologies [9, 10].

The fibrotic response may serve as a crucial reparative mechanism during episodes of acute kidney injury (AKI), promoting injury repair and extracellular matrix (ECM) remodeling [11, 12]. However, in cases of chronic injury, this delicate regulatory process is dysregulated, leading to the excessive accumulation of ECM that damages renal architecture and hampers kidney function [10, 13, 14]. Matrix metalloproteinases (MMPs), a family of 24 zinc-dependent endopeptidases, are key players in ECM dynamics, orchestrating ECM proteolysis and structural remodeling [15]. Besides their role in ECM degradation, MMPs modulate cellular behavior by cleaving bioactive molecules, thereby assuming context-specific functions in kidney homeostasis and pathology [16]. For example, acute upregulation of MMP-7 alleviates tubular apoptosis through Fas ligand cleavage and promotes regeneration by breaking down E-cadherin [17]. However, persistent activation of MMP-7 exacerbates tubulointerstitial fibrosis by degrading E-cadherin [18] and induces podocyte dysfunction through cleaving nephrin [19]. Similarly, MMP-2 and MMP-9 exhibit either renal protective or pro-fibrotic effects based on the specific disease context [20]. While MMP-2, MMP-7, and MMP-9 are extensively investigated in the pathogenesis of CKD, emerging studies have begun to illuminate a crucial role of MMP-10 in regulating kidney pathophysiology through mechanisms that go beyond traditional ECM regulation [21, 22].

As a secreted proteinase, MMP-10 belongs to the stromelysin subfamily of MMPs and possesses the ability to digest various ECM components, thereby modulating their compositions [23]. However, the diverse effects of MMP-10 are not only attributed to ECM turnover but also involve the degradation of other specific bioactive mediators. In acute injury scenarios, MMP-10 offers protection against acute lung [24] and skeletal muscle [25] injuries by modulating macrophage activity and chemokine expression. Conversely, chronic overexpression of MMP-10 leads to maladaptive remodeling and promotes inflammation [26], organ fibrosis [22, 27], atherosclerosis [28, 29], and tumor progression [30]. The expression of MMP-10 in normal kidneys is minimal, and its genetic deficiency does not trigger renal pathology, indicating that MMP-10 is dispensable for kidney homeostasis [31]. We recently showed that MMP-10 exerts a beneficial role by facilitating tubular cell repair and regeneration after AKI [21], whereas induction of MMP-10 aggravates podocyte injury, proteinuria and glomerulosclerosis in glomerular disease [22]. These observations indicate that the role of MMP-10 varies based on different diseases and the distinct stages of kidney injury. However, the role of MMP-10 in the evolution of kidney fibrosis in CKD remains unclear.

In the present study, we demonstrated that MMP-10 is upregulated in a variety of animal models of CKD and human kidney biopsies from CKD patients. We further showed that MMP-10 promotes kidney fibrosis by trans-activating β-catenin via the epidermal growth factor (EGF) receptor signaling. These findings offer novel insights into the mechanism by which MMP-10 is linked to the transactivation of β-catenin, a key profibrotic signaling in CKD.

## Results

### Upregulation of MMP-10 is a common feature in mouse and human CKD

**Fig. 1:**
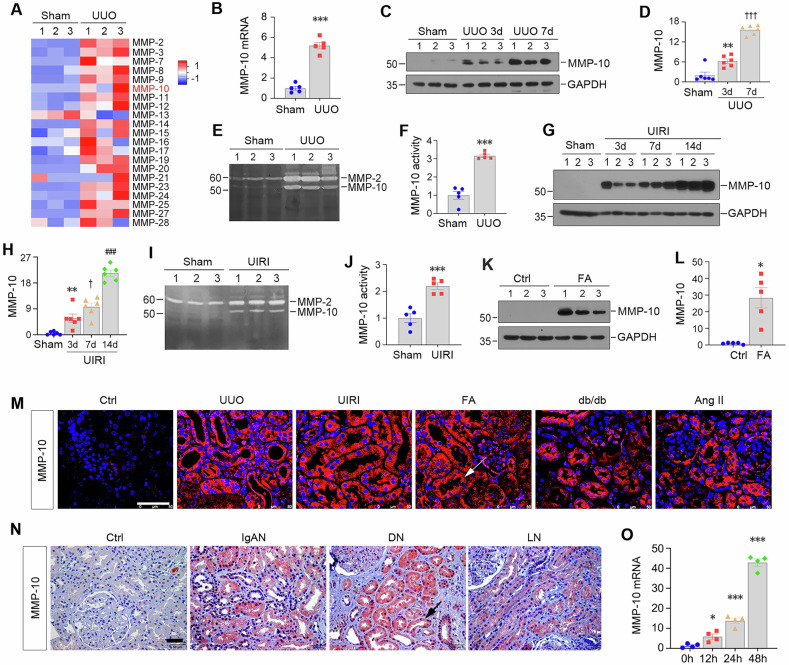
Matrix metalloproteinase-10 (MMP-10) is upregulated in kidney tubular epithelium after various injuries. **A** RNA sequencing revealed simultaneous induction of various MMPs in the kidney at 7 days after unilateral ureteral obstruction (UUO). Heat map depicts the mRNA expression profile of MMPs at 7 days after UUO. MMP-10 is highlighted by red color. **B** qRT-PCR showed renal MMP-10 mRNA expression at 7-day after UUO. Data are presented as the mean ± SEM. ***P < 0.001 versus the sham controls (*n* = 5). **C**, **D** Representative Western blotting (**C**) and quantitative data (**D**) showed the protein expression of MMP-10 in the kidney at different time points after UUO. Data are presented as the mean ± SEM. **P < 0.01 versus the sham controls (*n* = 6), ^†††^*P* < 0.001 versus the 3-day UUO (*n* = 6). **E**, **F** The gelatin zymography image (**E**) and quantitative data (**F**) showed an increased MMP-10 enzymatic activity. The band near 56 kDa was indicated as MMP-10. Data are presented as the mean ± SEM. ***P < 0.001 versus the sham controls (*n* = 5). **G, H** Representative Western blotting (**G**) and quantitative data (**H**) showed the induction of MMP-10 in the kidney at different time points after unilateral ischemia-reperfusion injury (UIRI). Data are presented as the mean ± SEM. **P < 0.01 versus the sham controls (*n* = 6), ^†^*P* < 0.05 versus the UIRI 3-day (*n* = 6), ^###^*P* < 0.001 versus the UIRI 7-day (*n* = 6). **I**, **J** Zymographic analysis further showed an increased proteolytic activity of MMP-10 in UIRI. Data are presented as the mean ± SEM. ***P < 0.001 versus the sham controls (*n* = 5). **K**, **L** Representative Western blotting (**K**) and quantitative data (**L**) showed renal induction of MMP-10 in folic acid-induced nephropathy (FA) at 14 days after a single intraperitoneal injection of 250 mg/kg. Data are presented as the mean ± SEM. *P < 0.05 versus the sham controls (*n* = 5). **M** Representative micrographs of the immunofluorescence staining for MMP-10 protein in various CKD models as indicated. The arrow indicates positive staining in renal tubules. Scale bar, 50 µm. **N** Representative micrographs of the immunohistochemical staining for MMP-10 in human kidney biopsies from various CKD as indicated. Arrow indicates positive staining. Scale bar, 50 µm. **O** Quantitative real-time PCR analysis (qRT-PCR) demonstrated the induction of MMP-10 mRNA in human proximal tubule cells (HK-2) after treatment with TGF-β1 (2 ng/ml). Data are presented as the mean ± SEM. *P < 0.05, **P < 0.01, ***P < 0.001 versus the controls (*n* = 4).

To study the regulation of MMPs in fibrotic kidneys, we employed an unbiased RNA-sequencing (RNA-seq) approach to profile their expression [32]. Figure 1A illustrates that the majority of MMP family members were upregulated in injured kidney at 7 days after unilateral ureteral obstruction (UUO), compared with sham controls. As MMP-10 was also upregulated in AKI and glomerular diseases [21, 22], we decided to focus our studies on this proteinase.

To validate the findings obtained from the RNA-seq data, we carried out experiments by using a mouse model of UUO. As shown in Fig. 1B, upregulation of MMP-10 mRNA was confirmed in the obstructed kidneys after UUO. Furthermore, a time-dependent upregulation of MMP-10 protein was observed at 3 and 7 days after UUO (Fig. 1C, D). Gelatin zymography further revealed an increased MMP-10 activity in the obstructed kidneys (Fig. 1E, F). To generalize this finding, we examined MMP-10 expression in other CKD models. Comparable induction of MMP-10 expression and activity was observed after unilateral ischemia-reperfusion injury (UIRI) (Fig. 1G–J). Similarly, marked induction of MMP-10 expression was also evident in animal model of folic acid-induced nephropathy (FAN) (Fig. 1K, L). To define MMP-10 localization, immunofluorescence staining was performed on several well-established CKD models, including UUO, UIRI, FAN, db/db, and angiotensin II (Ang II) infusion mice. Figure 1M revealed intense MMP-10 staining in the tubular epithelium of fibrotic kidneys, compared to normal controls. Co-staining with tubular segment-specific markers indicated a widespread MMP-10 expression across various tubular segments, including proximal tubules, distal tubules and collecting duct (Supplementary Fig. S1).

To investigate clinical relevance of these findings, we examined MMP-10 expression in kidney biopsies from CKD patients. Non-tumorous kidney tissue from renal cell carcinoma patients who underwent nephrectomy served as normal controls. As shown in Fig. 1N, MMP-10 protein was increased in renal tubules of CKD, including IgA nephropathy (IgAN), diabetic nephropathy (DN) and lupus nephritis (LN). In addition, incubation of human proximal tubular epithelial cells (HK-2) with TGF-β1 induced MMP-10 expression in vitro (Fig. 1O). These results suggest that induction of MMP-10 is a common pathological finding in mouse and human CKD.

### MMP-10 induces fibrotic response by activating β-catenin in vitro

**Fig. 2:**
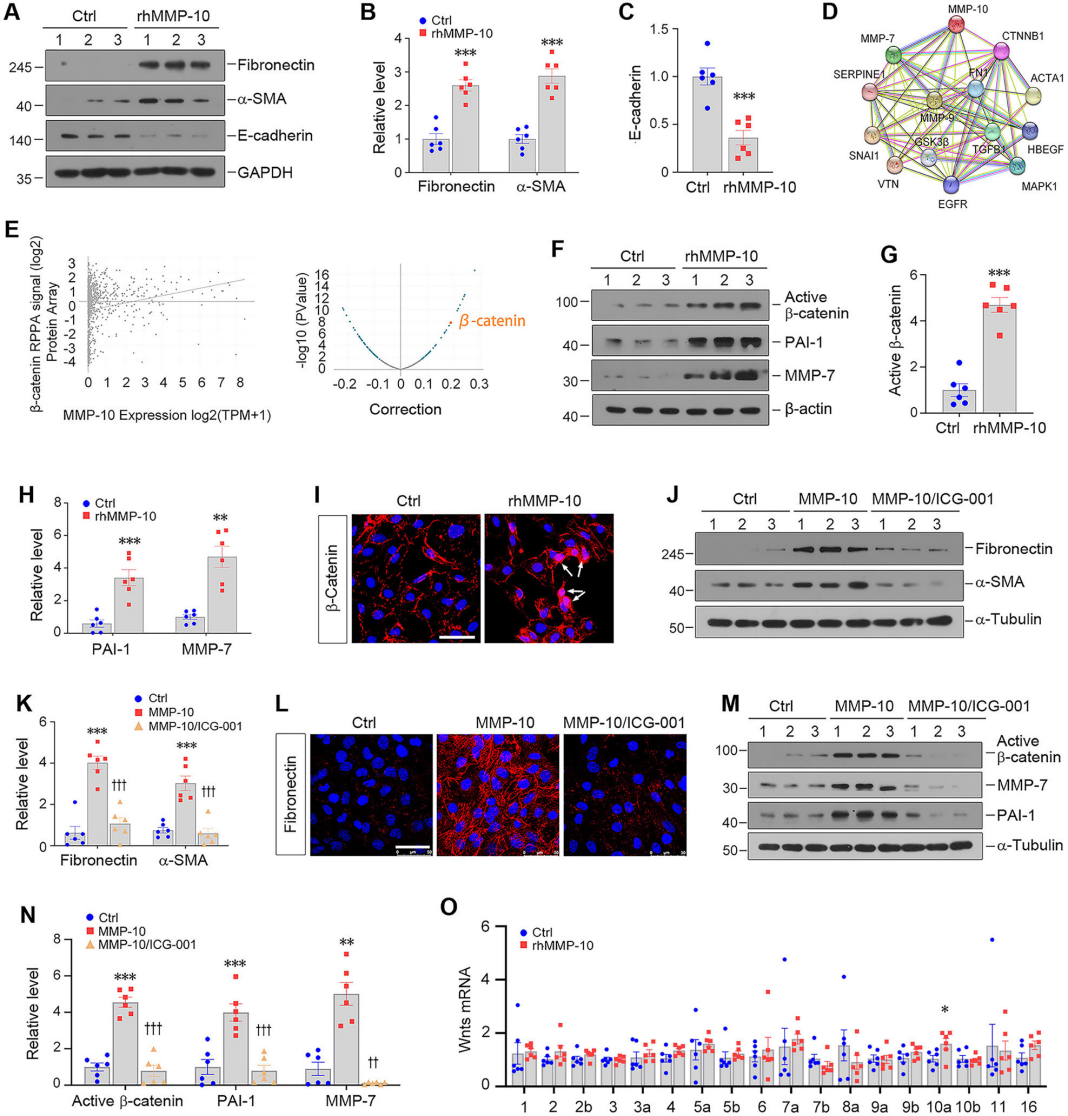
Identification of β-catenin as the major effector mediating MMP-10 action. **A**–**C** Western blotting showed that rhMMP-10 (100 ng/ml) induced fibronectin and α-smooth muscle actin (α-SMA) and repressed E-cadherin in HK-2 cells. Representative Western blotting (**A**) and quantitative data (**B**, **C**) are reported. Data are presented as the mean ± SEM. ***P < 0.001 versus the controls (*n* = 6). **D** STRING analysis revealed the protein-protein interaction (PPI) network involving MMP-10 (https://cn.string-db.org/, accessed on 8 August 2023). The interaction between MMP-10 and β-catenin was noticed. **E** Analysis of the Cancer Dependency Map (https://depmap.org, accessed on 10 August 2023) revealed the correlation between MMP-10 expression and alteration in β-catenin. Linear regression (left) and volcano plot (right) demonstrated a positive correlation between MMP-10 and β-catenin. **F, H** RhMMP-10 activated β-catenin signaling in vitro, leading to the induction of active β-catenin, PAI-1, and MMP-7 proteins. Data are presented as the mean ± SEM. **P < 0.01, ***P < 0.001 versus the controls (*n* = 6). **I** Representative micrographs showed the nuclear translocation of β-catenin after rhMMP-10 treatment for 12 h. White arrows indicate nuclear staining of β-catenin. Scale bar, 50 µm. **J**, **K** ICG-001 (10 μM) abolished MMP-10-mediated fibronectin and α-SMA expression. Data are presented as the mean ± SEM. ***P < 0.001 versus the controls (*n* = 6); ^†††^*P* < 0.001 versus rhMMP-10 alone (*n* = 6). **L** Representative immunofluorescence staining showed that ICG-001 inhibited fibronectin expression and deposition induced by MMP-10. Scale bar, 50 µm. **M**, **N** ICG-001 inhibited MMP-10-mediated β-catenin activation and its downstream MMP-7 and PAI-1 induction. Representative Western blotting (**M**) and quantitative data (**N**) were presented. Data are presented as the mean ± SEM. **P < 0.01, ***P < 0.001 versus the controls (*n* = 6); ^††^*P* < 0.01, ^†††^*P* < 0.001 versus rhMMP-10 alone (*n* = 6). **O** qRT-PCR analysis showed that rhMMP-10 did not significantly affect the expression of Wnt ligands except Wnt10a. Data are presented as the mean ± SEM. *P < 0.05 versus controls (*n* = 6).

Given the robust induction of MMP-10 in renal tubular cells in CKD, we sought to investigate its effect on tubular epithelial cells in vitro. HK-2 cells were stimulated with recombinant human MMP-10 (rhMMP-10) for 48 h. Western blotting revealed that rhMMP-10 induced fibronectin and α-smooth muscle actin (α-SMA) expression while suppressing E-cadherin (Fig. 2A–C). To elucidate the mechanism of MMP-10 actions, we constructed a protein-protein interaction (PPI) network using STRING analysis. As shown in Fig. 2D, there were intricate connections among MMP-10, β-catenin, MMP-7 and other fibrosis-related proteins. Furthermore, we analyzed the potential correlations of these proteins with MMP-10 expression by utilizing the Cancer Dependency Map, a comprehensive database for loss-of-function screening encompassing hundreds of cancer cell lines. Linear regression and volcano plot revealed a positive association between MMP-10 and β-catenin (Fig. 2E). These findings imply that β-catenin, a principal mediator of Wnt signaling and a crucial driver of CKD progression [33, 34], could serve as an effector mediating MMP-10 action.

We next experimentally validated this speculation by examining β-catenin activation by MMP-10. Figure 2F–H shows that treatment of HK-2 cells with rhMMP-10 induced β-catenin activation and upregulated its downstream targets plasminogen activator inhibitor-1 (PAI-1) and MMP-7 [35]. Consistently, immunofluorescence staining revealed nuclear translocation of β-catenin following rhMMP-10 incubation (Fig. 2I). Furthermore, the profibrotic effect of rhMMP-10 was abolished by ICG-001, a peptidomimetic inhibitor of β-catenin/CBP-mediated transcription [36] (Fig. 2J–L). ICG-001 also blocked the activation of β-catenin and induction of PAI-1 and MMP-7 by MMP-10 (Fig. 2M, N). However, except for Wnt10a, rhMMP-10 did not induce Wnt ligands in HK-2 cells (Fig. 2O), suggesting that MMP-10-triggered β-catenin activation is not primarily mediated by Wnt ligands.

### Knockdown of MMP-10 attenuates tubular injury and kidney fibrosis in vivo

**Fig. 3:**
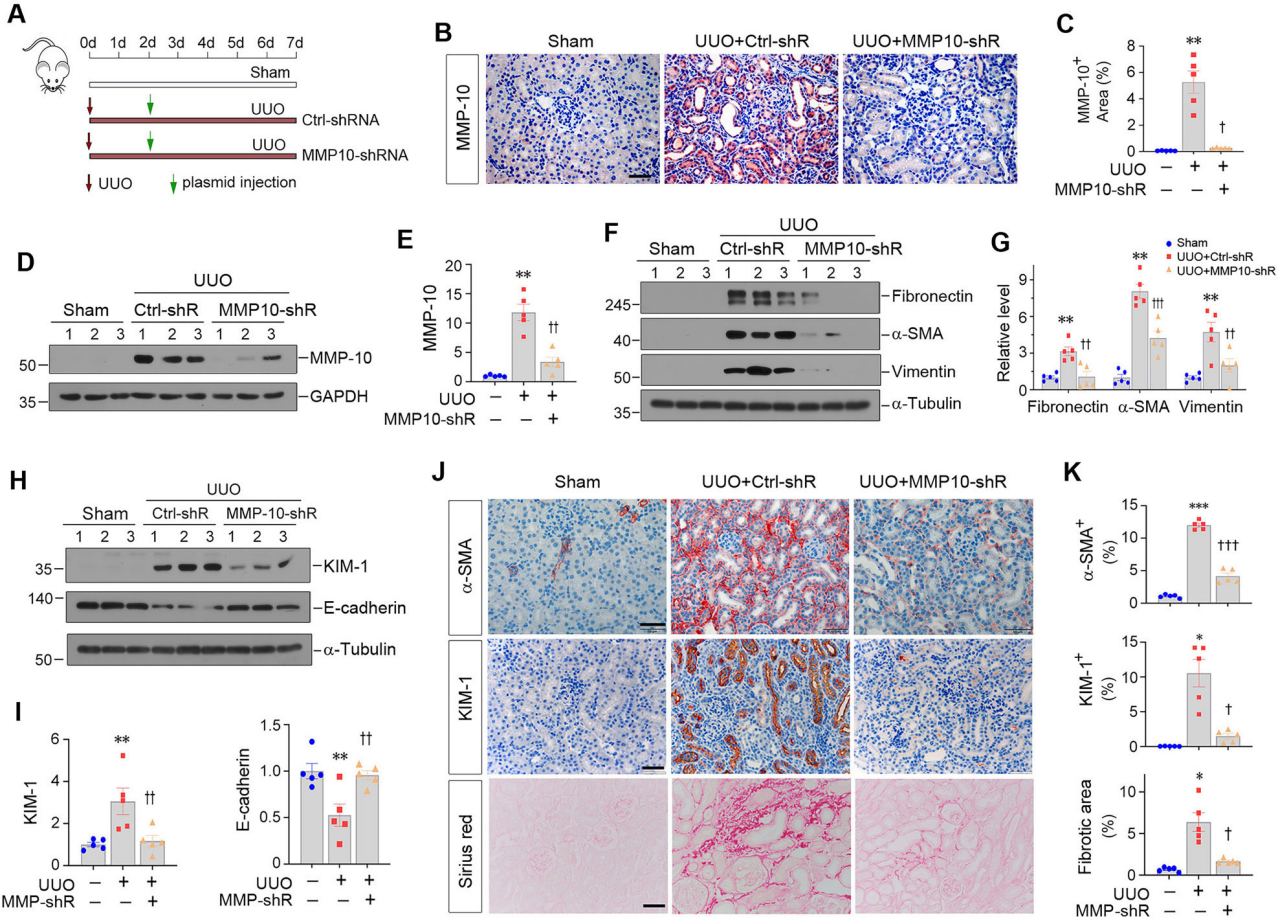
Knockdown of endogenous MMP-10 ameliorates kidney injury and fibrosis in obstructive nephropathy. **A** Experimental design. The red and green arrows show the timing of UUO surgery and plasmid injection, respectively. **B**, **C** Representative micrographs (**B**) and quantitative data (**C**) demonstrated MMP-10 protein expression in different groups as indicated. Scale bar, 50 µm. Data are presented as the mean ± SEM. **P < 0.01 versus sham controls (*n* = 5); ^†^*P* < 0.05 versus UUO (*n* = 5). **D**, **E** Western blot analysis showed MMP-10 protein levels in various groups as indicated. Data are presented as the mean ± SEM. **P < 0.01 versus sham controls (*n* = 5), ^††^*P* < 0.01 versus UUO (*n* = 5). **F**, **G** Western blot analysis showed the expression of several fibrosis-related proteins such as fibronectin, α-SMA, and vimentin. Representative Western blotting (**F**) and quantitative data (**G**) are presented. Data are presented as the mean ± SEM. **P < 0.01, ***P < 0.001 versus sham controls (*n* = 5); ^††^*P* < 0.01, ^†††^*P* < 0.001 versus UUO (*n* = 5). **H, I** Representative Western blotting (**H**) and quantitative data (**I**) showed KIM-1 and E-cadherin protein in various groups as indicated. Data are presented as the mean ± SEM. **P < 0.01 versus sham controls (*n* = 5), ^††^*P* < 0.01 versus UUO (*n* = 5). **J, K** Representative micrographs (**J**) and quantitative data (**K**) showed immunohistochemical staining for α-SMA and KIM-1 and Sirius red staining for collagen deposition. Data are presented as the mean ± SEM. *P < 0.05, **P < 0.01 versus sham controls (*n* = 5); ^†^*P* < 0.05, ^††^*P* < 0.01 versus UUO (*n* = 5). Scale bar, 50 µm.

To examine the role of MMP-10 in vivo, we utilized the kidney fibrosis model induced by UUO [37, 38]. Knockdown of endogenous MMP-10 in mice was carried out by intravenously injecting MMP-10-specific short hairpin RNA (shRNA) using a hydrodynamic-based gene delivery approach (Fig. 3A). Immunohistochemical staining confirmed a marked suppression of MMP-10 expression in the renal tubules of UUO mice injected with MMP-10-shRNA plasmid (Fig. 3B, C). Consistently, Western blot analysis revealed a decreased MMP-10 protein after shRNA-mediated inhibition (Fig. 3D, E).

We then investigated the effect of MMP-10 depletion on kidney fibrotic lesions. Figure 3F, G showed that knockdown of MMP-10 repressed renal expression of fibronectin, α-SMA, and vimentin after UUO. Furthermore, depletion of MMP-10 mitigated renal tubular injury and restored their epithelial phenotype, which was illustrated by repressing kidney injury molecule (KIM-1) and restoration of E-cadherin (Fig. 3H, I). Immunohistochemical staining for α-SMA and KIM-1 confirmed these findings (Fig. 3J, K). Furthermore, Sirius red staining demonstrated that inhibition of MMP-10 alleviated collagen accumulation and kidney fibrosis.

### MMP-10 transactivates β-catenin via HB-EGF/EGFR signaling

**Fig. 4:**
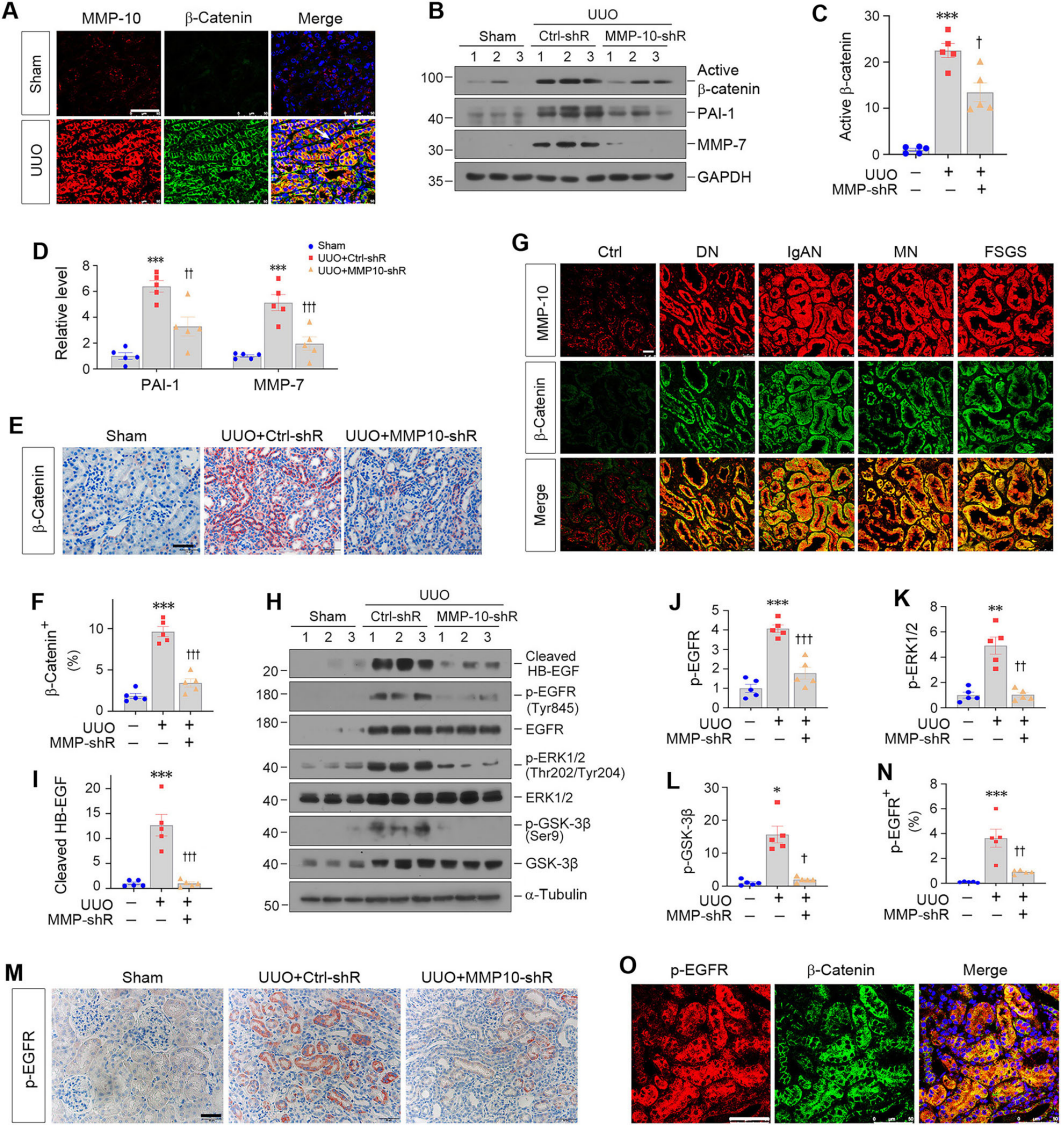
MMP-10 transactivates β-catenin via HB-EGF/EGFR/ERK1/2/GSK-3β cascade. **A** Representative micrographs showed the colocalization of MMP-10 (Red) and β-catenin (Green) in the obstructed kidney after UUO. Arrows indicate positive staining. Scale bar, 50 µm. **B**–**D** Knockdown of endogenous MMP-10 abolished the expression of β-catenin and its downstream genes in UUO model. Representative Western blotting results (**B**) and corresponding quantitative data (**C, D**) were shown. Data are presented as the mean ± SEM. ***P < 0.001 versus sham controls (*n* = 5); ^†^*P* < 0.05, ^††^*P* < 0.01, ^†††^*P* < 0.001 versus UUO injected with Ctrl-shRNA (*n* = 5). **E, F** Representative immunohistochemical staining for β-catenin further demonstrated that knockdown of MMP-10 inhibited β-catenin expression in renal tubules. Scale bar, 50 µm. Data are presented as the mean ± SEM. ***P < 0.001 versus sham controls (*n* = 5). ^†††^*P* < 0.001 versus UUO injected with Ctrl-shRNA (*n* = 5). **G** Double immunofluorescence staining showed the colocalization between MMP-10 (Red) and β-catenin (Green) in patients with various CKD as indicated. Scale bar, 25 µm. **H**–**L** Western blotting showed that knockdown of MMP-10 inhibited the expression of cleaved HB-EGF, p-EGFR (Tyr845), p-ERK (Thr202/Tyr204), and p-GSK-3β (Ser9) in the obstructed kidney after UUO. Representative Western blotting (**H**) and quantitative data (**I**–**L**) were shown. Data are presented as the mean ± SEM. *P < 0.05, **P < 0.01, ***P < 0.001 versus sham controls (*n* = 5); ^†^*P* < 0.05, ^††^*P* < 0.01, ^†††^*P* < 0.001 versus UUO injected with Ctrl-shRNA (*n* = 5). **M**, **N** Immunohistochemical staining for p-EGFR (Tyr845) demonstrated a decreased EGFR phosphorylation in renal tubules after MMP-10 knockdown. Scale bar, 50 µm. Data are presented as the mean ± SEM. ***P < 0.001 versus sham controls (*n* = 5), ^††^*P* < 0.01 versus UUO injected with Ctrl-shRNA (*n* = 5). **O** Representative micrographs showed the colocalization of p-EGFR (Red) and β-catenin (Green) in UUO. Scale bar, 50 µm.

We further investigated β-catenin activation by MMP-10 in vivo. As shown in Fig. 4A, co-expression of MMP-10 and β-catenin was observed in renal tubular epithelia of UUO mice. Comparable results were obtained in other CKD models induced by UIRI, FA or Ang II and in db/db mice (Supplementary Fig. S2A). Consistently, knockdown of MMP-10 repressed β-catenin activation and renal PAI-1 and MMP-7 expression in UUO mice (Fig. 4B–D). Immunohistochemical staining for β-catenin gave rise to similar results (Fig. 4E, F). Furthermore, co-localization of MMP-10 and β-catenin was validated in tubular epithelia of human kidney biopsies from patients with DN, IgAN, membranous nephropathy (MN) and focal and segmental glomerulosclerosis (FSGS) (Fig. 4G). These results reinforce the relationship between MMP-10 and β-catenin in the pathogenesis of human CKD.

To investigate the mechanism by which MMP-10 activates β-catenin, we studied the potential signaling pathways involved. As aforementioned, MMP-10 did not induce Wnt ligands (Fig. 2). Furthermore, MMP-10 failed to directly cleave E-cadherin (Supplementary Fig. S2B–E), a known regulator of β-catenin sequestration at the adherens junctions. Interestingly, knockdown of MMP-10 reduced the cleavage and activation of heparin-binding EGF-like growth factor (HB-EGF), thereby blocking EGFR phosphorylation at Try845 and subsequent downstream extracellular signal-regulated protein kinase-1 and -2 (ERK-1/2) activation at Thr202/Tyr204 (Fig. 4H–K). As phosphorylated ERK-1/2 inactivates glycogen synthase kinase 3β (GSK-3β) via phosphorylation [39], we observed reduced p-GSK-3β at Ser9 levels after MMP-10 inhibition in UUO kidney (Fig. 4H, L). These findings suggest that MMP-10 can induce β-catenin transactivation via the HB-EGF/EGFR/ERK/GSK-3β signaling cascade. In line with this notion, depletion of MMP-10 attenuated EGFR/ERK/GSK-3β pathway activation in UUO kidneys without affecting their total protein expression (Fig. 4H–L). Furthermore, depletion of MMP-10 reduced p-EGFR staining in renal tubules of UUO mice (Fig. 4M, N), and co-expression of p-EGFR and β-catenin was evident in tubular epithelia after UUO (Fig. 4O). These results demonstrate an intrinsic connection between EGFR activation and β-catenin signaling induced by MMP-10.

### Depletion of MMP-10 reduces kidney fibrosis after ischemic injury

**Fig. 5:**
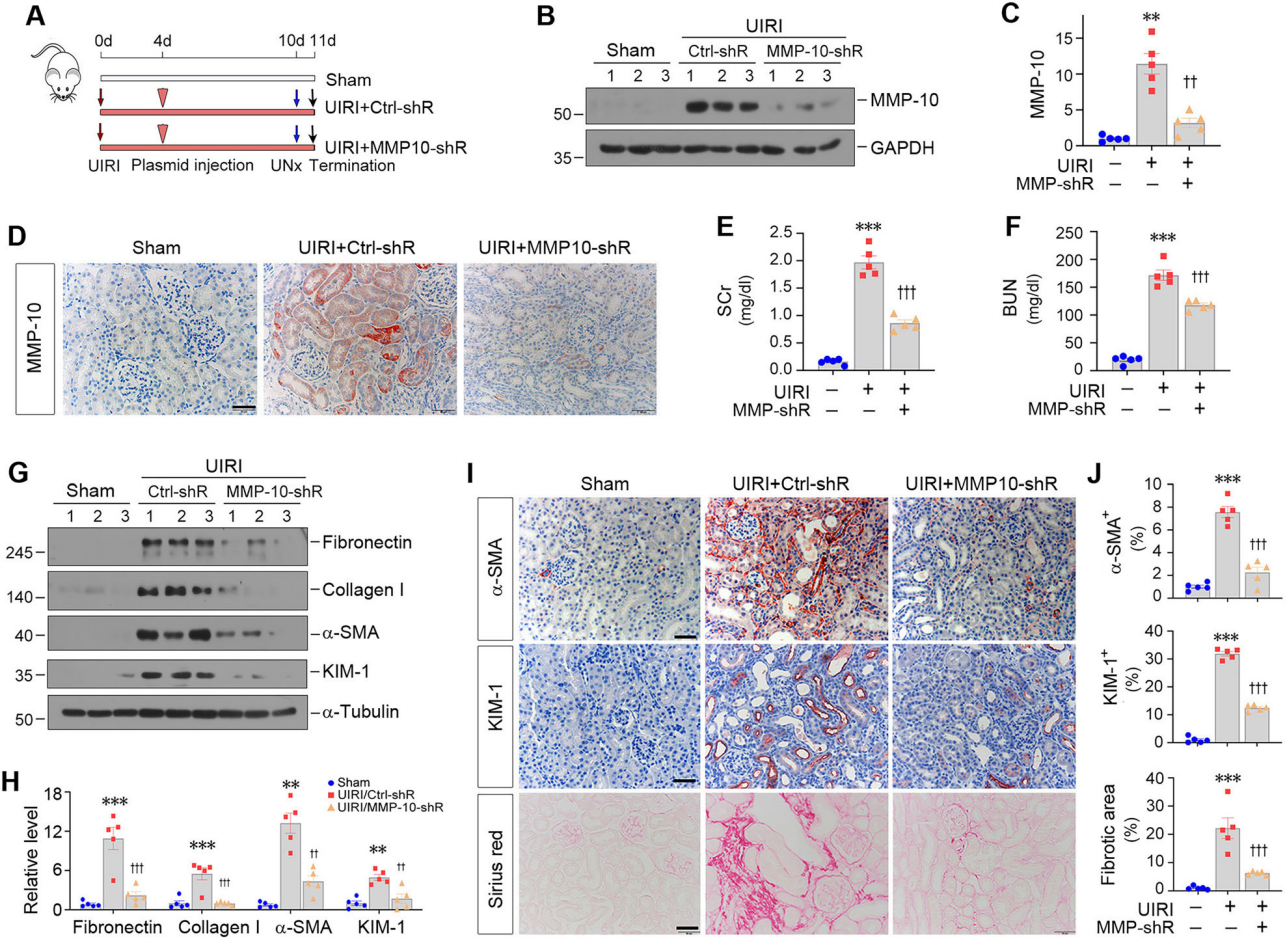
Depletion of endogenous MMP-10 reduces kidney injury and β-catenin activation after ischemia-reperfusion injury. **A** Experimental design. The timing of UIRI surgery, plasmid injection, unilateral nephrectomy, and sacrifice were indicated by red and black arrow or arrowhead as indicated, respectively. **B**, **C** Western blotting showed the protein level of MMP-10 in different groups as indicated. Representative Western blotting (**B**) and quantitative data (**C**) were shown. Data are presented as the mean ± SEM. **P < 0.01 versus sham controls (*n* = 5); ^††^*P* < 0.01 versus UIRI injected with Ctrl-shRNA (*n* = 5). **D** Representative images showed the expression and localization of MMP-10 in the kidney after different treatments. Scale bar, 50 µm. **E**, **F** Graphic presentation showed serum creatinine (SCr) and blood urea nitrogen (BUN) levels in three groups as indicated. Data are presented as the mean ± SEM. ***P < 0.001 versus sham controls (*n* = 5). ^†††^*P* < 0.001 versus UIRI injected with Ctrl-shRNA (*n* = 5). **G, H** Western blotting showed the protein levels of fibronectin, collagen I, α-SMA, and KIM-1 in different groups. Representative Western blotting (**G**) and quantitative data (**H**) were shown. Data are presented as the mean ± SEM. *P < 0.05, **P < 0.01, ***P < 0.001 versus sham controls (*n* = 5); ^†^*P* < 0.05, ^††^*P* < 0.01, ^†††^*P* < 0.001 versus UIRI injected with Ctrl-shRNA (*n* = 5). **I**, **J** Knockdown of MMP-10 ameliorated kidney injury and fibrosis. Renal fibrotic lesions were evaluated using Sirius red staining and immunohistochemical staining for α-SMA. The tubular injury was assessed through immunohistochemical staining for KIM-1. Scale bar, 50 µm. Data are presented as the mean ± SEM. ***P < 0.001 versus sham controls (*n* = 5), ^†††^*P* < 0.001 versus UIRI injected with Ctrl-shRNA (*n* = 5).

To generalize the findings of MMP-10 in promoting renal fibrosis, we administered MMP-10-specific shRNA or control shRNA to a UIRI-induced kidney fibrosis model, starting at 4 days after surgery (Fig. 5A). The efficacy of MMP-10 inhibition was confirmed by Western blot and immunohistochemical analyses (Fig. 5B–D). As shown in Fig. 5E, F, depletion of MMP-10 ameliorated kidney dysfunction in UIRI mice, as manifested by reduced SCr and BUN levels. Knockdown of MMP-10 also mitigated kidney injury and fibrotic lesions caused by UIRI (Fig. 5G, H). The expression of fibronectin, collagen I, α-SMA and KIM-1 proteins was reduced after MMP-10 depletion (Fig. 5G, H). Immunohistochemical staining for α-SMA and KIM-1 produced compatible results (Fig. 5I, J). Similarly, Sirius red staining exhibited a reduction in collagen deposition after MMP-10 depletion in UIRI mice.

We further investigated the effect of MMP-10 on the activation of both EGFR and β-catenin signaling. As shown in Supplementary Fig. S3A, B, knockdown of MMP-10 markedly inhibited β-catenin activation in UIRI mice. Meanwhile, depletion of MMP-10 effectively decreased cleaved HB-EGF and inhibited EGFR, ERK-1/2 and GSK3β phosphorylation. Furthermore, immunohistochemical staining confirmed a decreased p-EGFR staining in renal tubular epithelium (Supplementary Fig. S3C).

### EGFR activation is required for MMP-10-triggered β-catenin transactivation

**Fig. 6:**
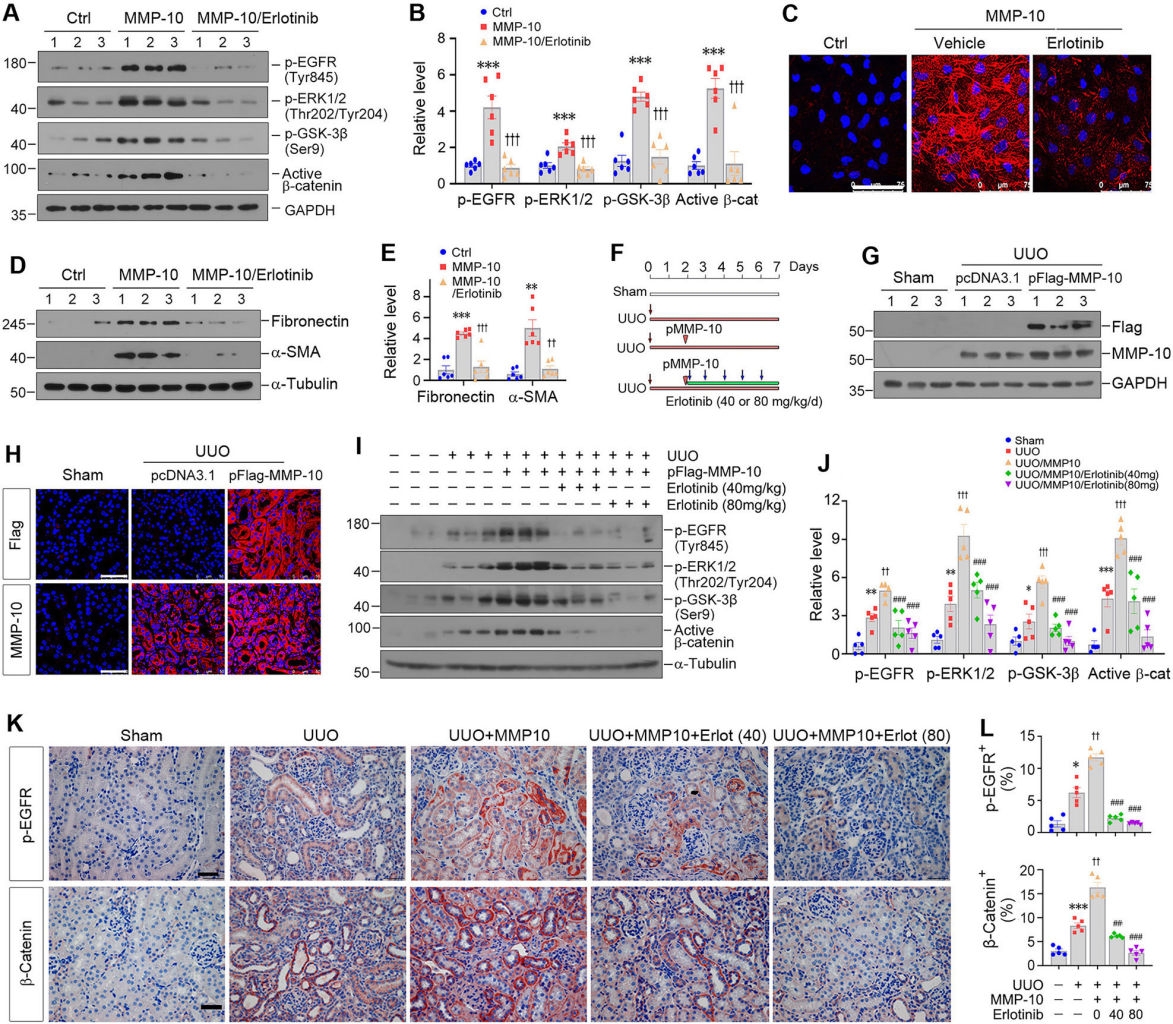
MMP-10-triggered β-catenin transactivation is dependent on EGFR signaling. **A**, **B** Blockade of EGFR signaling by small molecule inhibitor erlotinib abolished MMP-10-mediated β-catenin transactivation in vitro. Human proximal tubular epithelial cells (HK-2) were treated with rhMMP-10 in the absence or presence of erlotinib as indicated. Representative Western blotting (**A**) and quantitative data (**B**) showed the expression levels of p-EGFR (Tyr845), p-ERK1/2 (Thr202/Tyr204), p-GSK-3β (Ser9), and active β-catenin after various treatments. Data are presented as the mean ± SEM. ***P < 0.001 versus controls (*n* = 6); ^†††^*P* < 0.001 versus the group with rhMMP-10 treatment alone (*n* = 6). **C** Representative micrographs showed fibronectin expression and deposition in HK-2 cells after various treatments. Scale bar, 75 µm. **D**, **E** The expression of fibronectin and α-SMA was assessed in HK-2 cells after various treatments. Representative Western blotting (**D**) and quantitative data (**E**) were presented. Data are presented as the mean ± SEM. **P < 0.01, ***P < 0.001 versus controls (*n* = 6); ^††^*P* < 0.01, ^†††^*P* < 0.001 versus rhMMP-10 treatment alone (*n* = 6). **F** In vivo experimental design. The timing of UUO surgery, plasmid injection, and oral gavage of erlotinib at dose 40 mg/kg/d or 80 mg/kg/d is indicated, respectively. **G** Western blotting confirmed the expression of Flag and MMP-10 after Flag-tagged MMP-10 plasmid injection. **H** Representative micrographs also confirmed the expression and localization of Flag and MMP-10 in three groups as indicated. Scale bar, 50 µm. Representative Western blotting (**I**) and quantitative data (**J**) showed the expression levels of p-EGFR (Tyr845), p-ERK (Thr202/Tyr204), p-GSK-3β (Ser9), and active β-catenin in different groups. Data are presented as the mean ± SEM. *P < 0.05, **P < 0.01, ***P < 0.001 versus sham controls (*n* = 5); ^†^*P* < 0.05, ^††^*P* < 0.01, ^†††^*P* < 0.001 versus UUO plus pcDNA3.1 group (*n* = 5); ^#^*P* < 0.05, ^##^*P* < 0.01, ^###^*P* < 0.001 versus UUO plus pFlag-MMP-10 group (*n* = 5). **K**, **L** Representative micrographs (**K**) and quantitative data (**L**) showed immunohistochemical staining for p-EGFR (Tyr845) and β-catenin. Scale bar, 50 µm. Data are presented as the mean ± SEM. *P < 0.05, ***P < 0.001 versus sham controls (*n* = 5); ^††^*P* < 0.01 versus UUO plus pcDNA3.1 group (*n* = 5); ^##^*P* < 0.01, ^###^*P* < 0.001 versus UUO plus pFlag-MMP-10 group (*n* = 5).

To confirm the role of EGFR activation in mediating MMP-10-triggered β-catenin transactivation, we employed erlotinib, a highly selective EGFR tyrosine kinase inhibitor [40], in cultured HK-2 cells. As shown in Fig. 6A, B, erlotinib abolished the induction of p-EGFR, p-ERK1/2, p-GSK3β, and active β-catenin triggered by rhMMP-10. Notably, neither MMP-10 nor erlotinib affected total EGFR, ERK1/2, and GSK3β levels (Supplementary Fig. S4A). In addition, erlotinib abolished fibronectin expression and deposition induced by rhMMP-10, as illustrated by immunofluorescence staining (Fig. 6C). Western blotting also demonstrated that the induction of fibronectin and α-SMA was almost completely inhibited by erlotinib (Fig. 6D, E). Similar results were obtained when HK-2 cells were transfected with MMP-10 expression vector (Supplementary Fig. S4B–E).

We further investigated the effect of erlotinib in vivo. Figure 6F shows the detailed experimental protocol. For overexpression of exogenous MMP-10, mice were intravenously injected with the Flag-tagged MMP-10 expression vector (pFlag-MMP-10) via a hydrodynamic-based gene transfer approach. In addition, UUO mice overexpressing exogenous MMP-10 were treated with either vehicle or erlotinib at doses of 40 or 80 mg/kg/d, respectively (Fig. 6F). Western blotting and immunofluorescence staining demonstrated upregulated MMP-10 protein in the kidney of UUO mice injected with pFlag-MMP-10, compared to UUO alone (Fig. 6G, H, Supplementary Fig. S4F). Meanwhile, the group receiving pFlag-MMP-10 exhibited a specific Flag-MMP-10 fusion protein expression, which was undetectable in sham or UUO alone.

We found that exogenous Flag-MMP-10 expression in UUO mice augmented EGFR phosphorylation, which could be inhibited by erlotinib at doses of 40 or 80 mg/kg/d (Fig. 6I, J). This was substantiated by immunohistochemical staining for p-EGFR (Fig. 6K, L). Consistently, Flag-MMP-10 overexpression augmented ERK1/2 phosphorylation at Thr202/Tyr204 and GSK3β phosphorylation at Ser9, respectively (Fig. 6I, J). Furthermore, overexpression of MMP-10 also promoted the activation of β-catenin, compared to UUO alone. However, inhibition of EGFR by erlotinib repressed their activation. Immunohistochemical staining also demonstrated that erlotinib dose-dependently inhibited MMP-10-mediated EGFR and β-catenin activation (Fig. 6K, L). Collectively, these results suggest that EGFR activation is required for the transactivation of β-catenin triggered by MMP-10.

### EGFR activation plays an imperative role in mediating MMP-10-induced kidney fibrosis

**Fig. 7:**
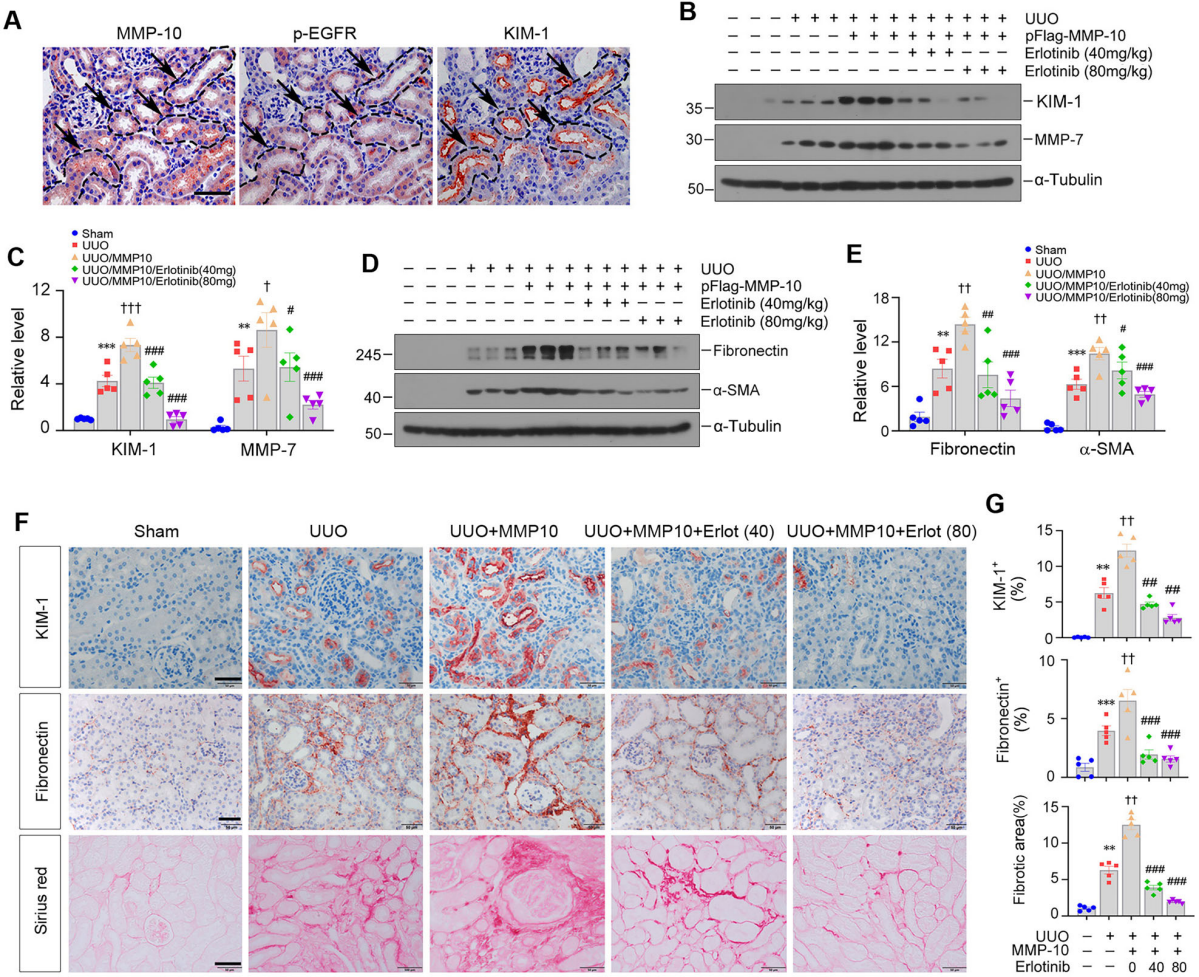
Inhibition of EGFR signaling blocks exogenous MMP-10-induced fibrotic lesions in UUO. **A** Immunohistochemical staining confirmed the co-localization of MMP-10, p-EGFR, and KIM-1 in serial sections of the obstructed kidney after UUO. Arrows indicate positive staining. Scale bar, 50 µm. **B**, **C** The expression levels of renal KIM-1 and MMP-7 in different groups were assessed by Western blotting. Data are presented as the mean ± SEM. **P < 0.01, ***P < 0.001 versus sham controls (*n* = 5); ^†^*P* < 0.05, ^†††^*P* < 0.001 versus UUO plus pcDNA3.1 group (*n* = 5); ^#^*P* < 0.05, ^###^*P* < 0.001 versus UUO plus pFlag^-^MMP-10 group (*n* = 5). **D**, **E** Representative Western blotting (**D**) and quantitative data (**E**) showed renal expression of fibronectin and α-SMA in different groups. Data are presented as the mean ± SEM. **P < 0.01, ***P < 0.001 versus sham controls (*n* = 5); ^††^*P* < 0.01 versus UUO plus pcDNA3.1 group (*n* = 5); ^#^*P* < 0.05, ^##^*P* < 0.01, ^###^*P* < 0.001 versus UUO plus pFlag-MMP-10 group (*n* = 5). **F**, **G** Representative micrographs (**F**) and quantitative data (**G**) showed renal expression of KIM-1 and fibronectin and fibrotic lesions by Sirius red staining. Scale bar, 50 µm. Data are presented as the mean ± SEM. **P < 0.01, ***P < 0.001 versus sham controls (*n* = 5); ^††^*P* < 0.01 versus UUO plus pcDNA3.1 group (*n* = 5); ^##^*P* < 0.01, ^###^*P* < 0.001 versus UUO plus pFlag-MMP-10 group (*n* = 5).

We next examined the efficacy of blocking EGFR activation in attenuating MMP-10-induced kidney fibrosis in vivo. Figure 7A revealed co-localization among MMP-10, p-EGFR and KIM-1 in fibrotic kidney, indicating a link between MMP-10-elicited EGFR activation and tubular injury. Accordingly, exogenous MMP-10 overexpression aggravated KIM-1 induction compared to UUO alone, while erlotinib counteracted the detrimental effect (Fig. 7B, C). In addition, MMP-10 induced MMP-7 expression, which was inhibited by erlotinib (Fig. 7B, C). Similarly, over-expression of MMP-10 augmented fibronectin and α-SMA expression, both of which were abolished by erlotinib (Fig. 7D, E). Immunohistochemical staining for KIM-1 and fibronectin, as well as Sirius red staining for collagen deposition gave rise to compatible results (Fig. 7F, G), suggesting that EGFR activation plays an indispensable role in mediating MMP-10-induced kidney fibrosis.

### MMP-10 and β-catenin constitute a positive feedback loop to promote kidney injury

**Fig. 8:**
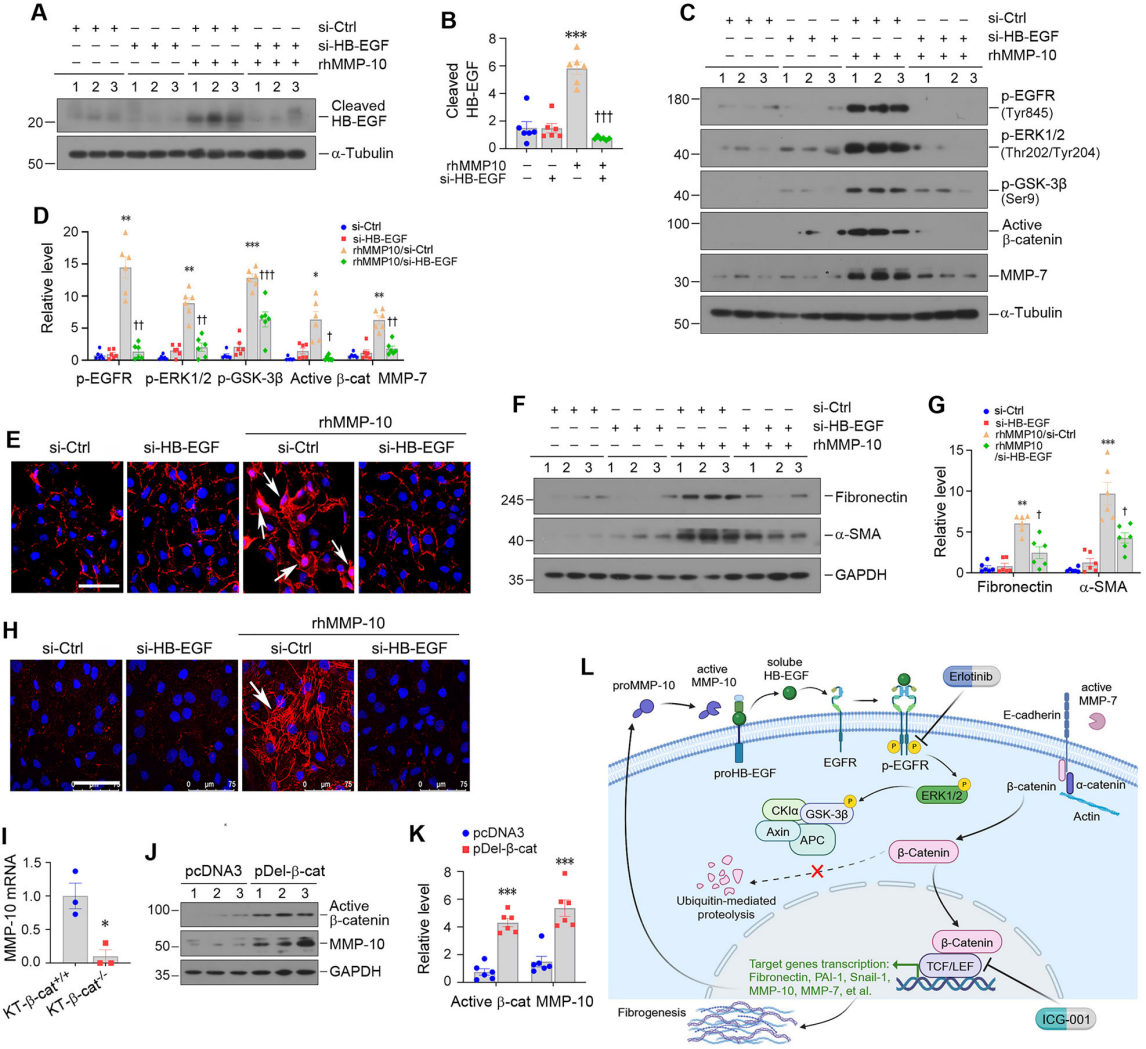
HB-EGF mediates MMP-10-triggered β-catenin transactivation. **A**, **B** Western blot analysis showed the expression of cleaved HB-EGF in HK-2 cells after various treatments. Data are presented as the mean ± SEM. ***P < 0.001 versus negative controls (*n* = 6); ^†††^*P* < 0.001 versus the group treated with rhMMP-10 (*n* = 6). **C**, **D** In vitro experiments showed that knockdown of HB-EGF effectively blocked rhMMP-10-induced expression of p-EGFR, p-ERK1/2, p-GSK-3β, active β-catenin and MMP-7. Data are presented as the mean ± SEM. *P < 0.05, **P < 0.01, ***P < 0.001 versus negative controls (*n* = 6); ^†^*P* < 0.05, ^††^*P* < 0.01, ^†††^*P* < 0.001 versus the group treated with rhMMP-10 (n = 6). **E** Representative micrographs demonstrated β-catenin activation and nuclear translocation induced by rhMMP-10, which was abolished by knocking down of HB-EGF. Scale bar, 75 µm. **F**, **G** Western blotting showed the expression of fibronectin and α-SMA in HK-2 cells after various treatments as indicated. Knockdown of HB-EGF abolished rhMMP-10-induced fibronectin and α-SMA expression. Data are presented as the mean ± SEM. **P < 0.01, ***P < 0.001 versus controls (*n* = 6); ^†^*P* < 0.05 versus the group treated with rhMMP-10 (*n* = 6). **H** Representative immunofluorescence images illustrated the expression levels of fibronectin across all treatment groups. Scale bar, 75 µm. **I** Re-analysis of an independent study through data mining (GSE193282) revealed that knockout of tubular β-catenin in mice abolished renal MMP-10 expression after UUO. Data are presented as the mean ± SEM. *P < 0.05 versus wild-type controls (*n* = 3). **J**, **K** Transient transfection with a N-terminus-truncated, constitutively activated β-catenin expression vector (pDel-β-cat) induced MMP-10 expression in HK-2 cells. Data are presented as the mean ± SEM. ***P < 0.001 versus pcDNA3 controls (*n* = 6). **L** Schematic diagram depicts the potential mechanism of MMP-10 action in kidney fibrosis. Upregulated MMP-10, as a response to injury, cleaves the HB-EGF, leading to its activation, which then activates EGFR and subsequently phosphorylates ERK1/2 and GSK-3β. This cascade of event causes β-catenin accumulation and translocation into the nucleus. Intranuclear β-catenin binds to TCF/LEF and promotes the transcription of its target genes including MMP-10, forming a vicious cycle. In addition, MMP-10 induces the expression of MMP-7 via β-catenin, which enzymatically degrades E-cadherin, liberating more β-catenin and further promoting kidney fibrosis.

To further study the interplay between MMP-10 and β-catenin, we knocked down HB-EGF expression in HK-2 cells using specific siRNA (si-HB-EGF) and explored its role in mediating MMP-10 action. Figure 8A, B showed that rhMMP-10 upregulated the cleaved form of HB-EGF, which was abolished by transfecting si-HB-EGF. Furthermore, knockdown of HB-EGF blocked the sequential phosphorylation of EGFR, ERK1/2, and GSK-3β induced by MMP-10 (Fig. 8C, D). Moreover, transactivation of β-catenin signaling caused by rhMMP-10 was also inhibited upon HB-EGF depletion, as evidenced by the inhibition of β-catenin activation and MMP-7 induction (Fig. 8C, D). Immunofluorescence staining revealed that knockdown of HB-EGF blocked the nuclear translocation of β-catenin after rhMMP-10 treatment (Fig. 8E). Accordingly, depletion of HB-EGF also abolished rhMMP-10-induced fibronectin and α-SMA expression in HK-2 cells (Fig. 8F, G), a finding corroborated by immunofluorescence staining (Fig. 8H).

The *MMP-10* promoter harbors two TCF/LEF-binding elements [41], suggesting that MMP-10 itself may be subjected to β-catenin regulation. To explore this possibility, we investigated the role of β-catenin in MMP-10 regulation in diseased kidneys. Data-mining and bioinformatics analysis revealed that tubule-specific ablation of β-catenin markedly downregulated MMP-10 expression after UUO (Fig. 8I). Furthermore, overexpression of constitutively activated β-catenin (pDel-β-cat) induced MMP-10 expression in HK-2 cells (Fig. 8J, K). Therefore, it appears clear that MMP-10 and β-catenin mutually stimulate each other, and together they constitute a positive feed-forward loop to promote kidney injury and fibrosis (Fig. 8L).

## Discussion

In this study, we show that almost all of MMPs are differentially upregulated in the fibrotic kidneys after UUO, suggesting a coherent and common response after tissue injury. Of particular interest, MMP-10 is induced in all kidney fibrosis models tested, such as UUO, UIRI, FAN, db/db mice, and Ang II-infusion, as well as in human kidney biopsies from patients with various CKD, such as IgA nephrology, diabetic nephrology and lupus nephritis (Fig. 1). MMP-10 is predominantly localized in kidney tubular epithelium, which constitutes the largest proportion of renal parenchyma and represents the primary target of injury [42, 43]. We further show that MMP-10 activates EGFR through shedding HB-EGF ectodomain and subsequently triggers transactivation of β-catenin. These studies establish a critical role for MMP-10 in the pathogenesis of kidney fibrosis by trans-activating β-catenin via HB-EGF/EGFR signaling. Because activated β-catenin in turn induces MMP-10 expression, our findings also suggest that MMP-10 and β-catenin constitute a vicious cycle that promotes kidney injury and fibrosis (Fig. 8L).

Although MMP-10 is kidney protective in AKI [21], its role in the pathogenesis of kidney fibrosis in CKD was unexplored. Functionally, beyond its involvement in ECM turnover, MMP-10 triggers a complex cascade of cellular events by cleaving non-ECM substrates, acting as a double-edged sword in response to environmental stress [23]. MMP-10 is essential for tissue repair during early injury phages in organs such as the kidney, lung, skin and skeletal muscle [21, 24, 25]; however, its dysregulation also implicate in various diseases [22, 26, 27, 29], in which high levels are often correlated with worse outcomes [22, 30]. Our findings in the present study provide clear evidence that MMP-10 is dysregulated in a wide variety of CKD in animal models and human kidney biopsies. Furthermore, over-expression of exogenous MMP-10 aggravates kidney injury and fibrosis, whereas knockdown of endogenous MMP-10 ameliorates fibrotic lesions (Figs. 3–6). Collectively, these results illuminate that contrary to its protective action in AKI, MMP-10 actually plays a detrimental role promoting renal fibrogenesis in the setting of CKD.

The present study uncovers that MMP-10 acts as a potent upstream regulator of β-catenin, driving the progression of tubulointerstitial fibrosis. As the principal intracellular mediator of Wnt signaling, β-catenin transduces its signals by upregulating Wnt target genes through interacting with T-cell factor/lymphoid enhancer-binding factor (TCF/LEF) [35, 44]. It should be stressed that activation of β-catenin in tubular epithelial cells by MMP-10 not only elicits its effect on tubular cells themselves, but also mediates interstitial fibroblast activation [45] and contributes to glomerular injury through an indirect paracrine fashion [19, 35]. Through an integrated bioinformatics analysis and experimental validation, we have elucidated that MMP-10 induces the nuclear translocation of β-catenin and upregulates its downstream PAI-1 and MMP-7. Consistently, ICG-001, an inhibitor that selectively blocks β-catenin signaling [46], can mitigate the profibrotic effects of MMP-10. These studies establish an intimate connection between MMP-10 induction and β-catenin activation in fibrotic kidneys.

It is well known that β-catenin also plays a role in forming adherent junction complexes. Cytoplasmic β-catenin is recruited to the cell membrane where it interacts with the intracellular tail structure of E-cadherin, forming a cell adhesion complex [47]. As such, the cleavage of E-cadherin, facilitated by several MMPs including MMP-3, MMP-7, and MMP-9, leads to the liberation of β-catenin [18, 48, 49]. Despite the high sequence similarity in the catalytic domains of MMP-3 and MMP-10 [50], our results indicate that E-cadherin is not a direct target of MMP-10 (Supplementary Fig. S2). However, we observed that MMP-10 can trigger an increase in MMP-7 expression in HK-2 cells. Therefore, possibility still exists that MMP-10 may also activate β-catenin signaling by an alternative route via triggering MMP-7-mediated E-cadherin degradation (Fig. 8L).

The primary source of β-catenin for signal transduction arises from the cytoplasmic pool of liberated β-catenin, which is subject to phosphorylation and degradation by the so-called destruction complexes regulated by classical Wnt ligands [34, 51]. However, exposure to rhMMP-10 in vitro does not significantly affect the expression of Wnt ligands, suggesting the potential involvement of alternative mechanisms. Consistent with previous findings showing that MMP-10 can enhance the expression of phosphorylated GSK-3β [52], a critical component of the destruction complex, our data strongly support the notion that MMP-10 activates β-catenin by affecting the functionality of the destruction complex. Specifically, MMP-10-mediated cleavage of HB-EGF activates EGFR, leading to ERK-1/2 phosphorylation, GSK-3β inactivation and β-catenin activation. This cascade is essential for MMP-10-triggered transactivation of β-catenin signaling. Therefore, our results provide unambiguous evidence that MMP-10 can activate β-catenin signaling through the HB-EGF/EGFR/ERK/GSK-3β cascade.

It is interesting to note that there are two putative TCF/LEF binding sites within the *MMP-10* promoter [41, 53], implying MMP-10 as a potential target of active β-catenin. Indeed, data mining unveils that specific ablation of β-catenin in renal tubular epithelial cells abolishes MMP-10 expression in the kidney after UUO. Furthermore, studies conducted on HK-2 cells demonstrate that activation of β-catenin induces MMP-10 expression, aligning with a previous report that GADD45a upregulates MMP-10 via the GSK-3β/β-catenin signaling [53]. Likewise, inhibition of either MEK1/2 or GSK-3β eradicates WISP3/CCN6-mediated MMP-10 expression [54]. In line with these findings, our results suggest a plausible mechanism in which MMP-10-transactivated β-catenin triggers the subsequent transcription of MMP-10 itself, perpetuating renal fibrotic lesions.

In summary, we show in this study that upregulation of MMP-10 is a common and coherent response in kidney tubular epithelial cells after injury, which drives the development and progression of renal fibrosis. Mechanistically, MMP-10 promotes kidney fibrosis through transactivating β-catenin in a Wnt-independent fashion, which involves a cascade of events encompassing HB-EGF, EGFR, ERK and GSK-3β. Although more studies are needed, our findings provide a compelling rationale for targeting MMP-10 as a novel approach to ameliorate fibrotic CKD.

## Materials and methods

### Animal models

Wild-type male C57BL/6 mice (6–8 weeks old) were purchased from Vital River Laboratory (Beijing, China) and housed under standardized conditions at the Experimental Animal Center of Nanfang Hospital. Group randomization was performed using the online tool “Research Randomizer” (https://www.randomizer.org). Each group consisted of five or six mice, which satisfied the minimum sample size requirement for conducting an Analysis of Variance (ANOVA) test. All animals were included in the analysis. To investigate MMP-10 expression in the kidneys across various CKD models, we employed five established protocols: unilateral ureteral obstruction (UUO), unilateral ischemia reperfusion injury (UIRI), folic acid-induced nephropathy (FAN), diabetic *db/db* mice, and chronic Ang II infusion [36, 55–57]. For the UUO model, following midline laparotomy under general anesthesia, the left ureter was ligated with 4-0 silk. Kidneys were collected 7 days post-procedure for analysis [56]. For the UIRI model, unilateral ischemia was induced by clamping the left renal pedicle for 35 min, with body temperature maintained at 37.5 °C during surgery. Contralateral nephrectomy was performed on day 10, followed by tissues collection on day 11 [55]. For the FAN model, a single intraperitoneal injection of folic acid (250 mg/kg) (F7876; Merk, Darmstadt, DE) was administered, and kidneys were collected 14 days post-injection [57]. For the Ang II infusion model, osmotic minipumps (Model 2ML4; Alzet) were subcutaneously implanted to continuously deliver Ang II (0.75 mg/kg/d) (4006473.0100100010781; BACHEM, Bubendrof, CH) for 4 weeks prior to kidneys collected [36]. The diabetic *db/db* mice were purchased from Cavens Model Animal Company (Jiangsu, China).

To clarify the function of MMP-10, three sets of animal experiments were conducted. Figures 3A, 5A and 6F depict the complex experimental designs. Forced overexpression of exogenous *MMP-10* gene or knockdown of endogenous MMP-10 in vivo was carried out by hydrodynamic-based gene delivery methods at 1 mg/kg [22, 56]. MMP-10 knockdown was performed by shRNA technology, in which the MMP-10 siRNA sequence (5′CCAGCTAACTTCCACCTTT3′) was incorporated into the MMP-10 shRNA plasmid vector. Erlotinib (Cat. HY-50896; MedChemExpress, New Jersey, USA) was administered by oral gavage at doses of 40 mg/kg/day or 80 mg/kg/day for 5 days, as indicated in Fig. 6F.

### Human kidney biopsies

Human kidney biopsies were obtained from diagnostic renal biopsies performed at Nanfang Hospital, with written informed consent from the patients. The normal control sections were obtained from non-tumorous kidney tissues of the patients with renal cell carcinoma. The use of human kidney specimens in this study was approved by the institutional Ethics Committee at Nanfang Hospital. The renal biopsy tissue is embedded in paraffin and sectioned for subsequent immunostaining experiments.

### Cell culture and treatment

Human kidney proximal tubular cells (HK-2) were purchased from the Cell Bank of the Chinese Academy of Science (Shanghai, China) and cultured in DMEM/ Ham’s F12 medium supplemented with 10% fetal bovine serum. The cells were identified by STR profiling and confirmed to be free of mycoplasma contamination. Cells from the same passage (P3-P5) were then plated into 6-well plates. Following a 12-h serum deprivation, the cells were randomized into experimental groups (*n* = 6 biological replicates per group) using the “Research Randomizer” online tool (https://www.randomizer.org). HK-2 cells were pretreated with 10 μM ICG-001 (Cat. HY-14428, MedChemExpress) or 2 μM erlotinib (Cat. HY-50896, MedChemExpress) for 1 h, followed by exposure to 100 ng/ml recombinant human MMP-10 (rhMMP-10, Cat. 910-MP-010, R&D Systems, Minnesota, USA) for 48 h. In some experiments, HK-2 cells were transiently transfected with plasmid vectors or siRNA, such as pFlag-MMP-10, pDel-β-cat or siHB-EGF, according to established procedures [36], and subsequently incubated with indicated reagents for an additional 48 h.

### Western blot analysis

Western blotting was performed according to a standardized protocol [36]. Briefly, protein from mouse kidney homogenates or cell lysates were quantified using the BCA Protein assay Kit (Cat. ab287853; Abcam, Cambridge, UK). For each lane, 20-40 µg of protein was separated by SDS-PAGE and transferred to PVDF membranes (Cat. IPVH00010, MilliporeSigma). Membranes were then blocked with 5% non-fat milk and incubated overnight at 4 °C with the respective primary antibodies as listed in Supplementary Table S1. Membranes were then incubated with HRP-conjugated secondary antibodies, followed by protein band visualization using SuperEnhanced chemiluminescence reagents (Cat. P1010, Applygen Technologies Inc, Beijing, China) and imaging on Kodak X-ray film. Membrane stripping was performed with Restore PLUS buffer (Cat. 46430, Thermo Fisher Scientific, Massachusetts, USA) before re-probing with antibodies targeting housekeeping proteins. Densitometric quantification was carried out with ImageJ software (NIH), and signal intensities were normalized to loading controls (GAPDH, α-tubulin, or β-actin). Relative protein expression levels were expressed as fold changes compared to the control group (set as 1.0).

### RNA extraction and quantitative real-time RT-PCR

Total RNA was extracted using the TRIzol RNA isolation reagent (Thermo Fisher Scientific). First-strand cDNA synthesis was performed with the Reverse Transcription System kit (Promega) as previously described [36]. Quantitative real-time RT-PCR (qRT-PCR) was conducted on the ABI PRISM 7000 Sequence Detection System (Applied Biosystems, Foster City, CA) according to standard protocols [58]. Primer sequences for qRT-PCR are listed in Supplementary Table S2. The mRNA expression levels were normalized to β-actin using the ΔΔCT method to facilitate precise quantification and comparative analysis.

### Gelatin zymography

MMP-10 enzymatic activity in kidney tissue extracts was assessed using Gelatin zymography, according to the method reported previously [22]. Whole kidney tissues were homogenized in 1× PBS, centrifuged at 4 °C, 12,500 rpm for 10 min, and the supernatant was collected. Protein concentrations were assessed using the BCA assay. Samples were combined with 5× non-reducing sample buffer (4% SDS, 20% glycerol, 0.01% bromophenol blue, 125 mM Tris-HCl, pH 6.8) and subjected to electrophoresis on an 8% acrylamide gel containing 0.2% (w/v) gelatin (Cat. ST1339, Beyotime, Shanghai, China). After electrophoresis, the gel was washed twice for 30 min with a washing buffer (2.5% Triton X-100, 50 mM Tris-HCl, pH 7.5, 5 mM CaCl_2_, 1 μM ZnCl_2_), followed by a rinse in an incubation buffer (1% Triton X-100, 50 mM Tris-HCl, pH 7.5, 5 mM CaCl2, 1 μM ZnCl2) at 37 °C with agitation. The gel was then incubated for 24 h at 37 °C in fresh incubation buffer. Proteolytic activity was visualized after staining with Coomassie Blue and destaining with a solution (40% methanol, 10% acetic acid, 50% H_2_O), revealing MMP-10 activity as colorless bands on a blue background.

### Determination of serum creatinine and BUN

The levels of serum creatine and BUN were assessed using an automated chemistry analyzer (AU480; Beckman Coulter Inc; Kraemer Boulevard Brea, CA). The concentrations of serum creatinine and BUN were reported as milligrams per deciliter (mg/dl).

### Assessment of E-cadherin degradation by MMP-10

Immunoprecipitation was performed using 500 µg of NP-40 lysed HK-2 cell protein and 1 µg of anti-E-cadherin antibodies (Cat. 14472S, Cell Signal Technology, Massachusetts, USA) according to standard protocol [59]. The immunoprecipitated complexes were resuspended in a buffer containing of 50 mM Tris-Hcl (pH 7.5), 10 mM CaCl_2_, 150 mM NaCl, 0.05% Brij­35, followed by incubation with 200 ng of recombinant human MMP-10 for the time periods specified in Supplementary Fig. S2D, E. The samples were subsequently subjected to conventional Western blot analysis with an anti-E-cadherin antibody (Cat. Ab76055; Abcam).

### Histology, immunohistochemical and immunofluorescence staining

Paraffin-embedded sections of mouse and human kidney (3 µm thickness) were processed according to standard protocols. Mouse kidney sections were subjected to Sirius red staining for collagen quantification and immunostaining for specific proteins localization, as detailed previously [36]. Immunofluorescence staining was conducted on cultured cells or frozen mouse kidney sections using established procedures [18], with images captured under Confocal microscopy. The corresponding primary antibodies utilized are listed in Supplementary Table S1.

The fibrotic lesion and positive area quantification were performed using a computerized point-counting method. For each kidney section, approximately 10 non-overlapping 40× cortical images were randomly selected and captured under an Olympus light microscope. Fibrotic lesions and positive area, as well as total field area measurements, were conducted using Image-Pro Plus software (Media Cybernetics). The percentage of fibrotic lesions or positive areas was calculated with respect to the total area. Data for each mouse represent the mean of the measurements from the 10 images.

### Bioinformatic analysis

The RNA-seq-derived MMPs expression heatmap was generated via Heatmapper (http://heatmapper.ca) based on Fragments Per Kilobase Million (FPKM) values (National Center Biotechnology Information, PRJNA846588) [32]. MMP-10 expression levels in tubular epithelia-specific β-catenin knockout (β-catenin-/-) mice subjected to UUO were quantified as FPKM values normalized to wild-type controls (GSE193282) [45]. The protein interaction network conducted using STRING v12.0 (http://string-db.org, accessed on 8 August 2023). Functional relationships between MMP-10 and β-catenin were analyzed using Cancer Dependency Map (https://depmap.org, accessed on 10 August 2023).

### Statistical analyses

All quantitative data are presented as mean ± standard error of the mean (SEM). Histological quantitation was performed by researcher who was blinded to treatment. Statistical analysis was conducted using SPSS 25.0 (SPSS Inc, lllinois, USA), while data visualization was performed using GraphPad Prism 8 (GraphPad Software, California, USA). Comparisons between two groups were evaluated by a two-side independent-sample t-test. For multiple group comparisons, one-way analysis of variance (ANOVA) was employed, followed by the Fisher’s least significant difference (LSD) test when equal variances were confirmed by Levene’s test. In case of variance heterogeneity, Dunnett’s T3 test were implemented. Statistical significance was defined as *P*-values < 0.05. Representative results shown in figures were derived from at least three independent biological replicates.

## Supplementary information

Supplementary material

Original Western blots

## Data Availability

The raw data and materials supporting the conclusions of this study are available from the corresponding authors upon request.
