# Supplementary material for: Matrix metalloproteinase-10 promotes kidney fibrosis by transactivating β-catenin signaling
Source: Cell Death Discov. 2025 May 17;11:241. doi: 10.1038/s41420-025-02521-w (PMC12085647; doi:10.1038/s41420-025-02521-w)
Supplement: Supplementary file 1 — Supplementary material [file 41420_2025_2521_MOESM1_ESM.pdf]

Matrix metalloproteinase-10 promotes kidney fibrosis by transactivating β-catenin signaling

Xiaoli Sun, Qian Ren, Xi Liu, Huishi Tan, Zhanji Zhan, Enqing Lin, Yinyi Long, Xue Hong, Lili Zhou and Youhua Liu

SUPPLEMENTARY FIGURES AND TABLES

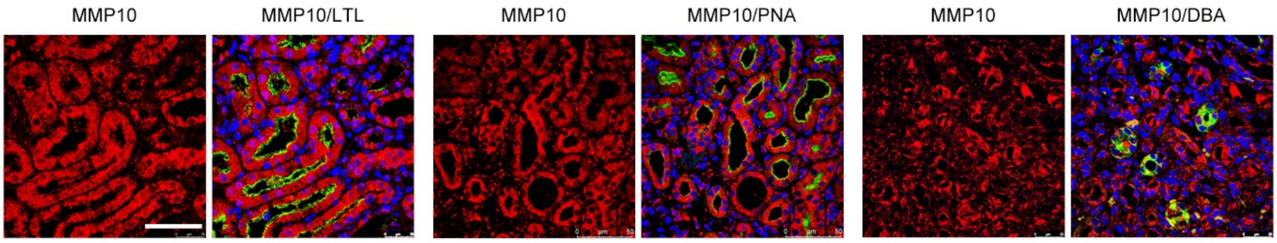

Supplementary Figure S1. Localization of MMP-10 in the kidney subjected to unilateral ureteral obstruction (UUO). In mouse UUO kidney, colocalization of MMP-10 (Red) and various segment-specific renal tubular markers (Green), including proximal tubule marker lotus tetragonolobus (LTL), distal tubule marker peanut agglutinin (PNA), and collecting duct marker dolichos biflorus agglutinin (DBA), could be observed. Scale bar, 50 μm.

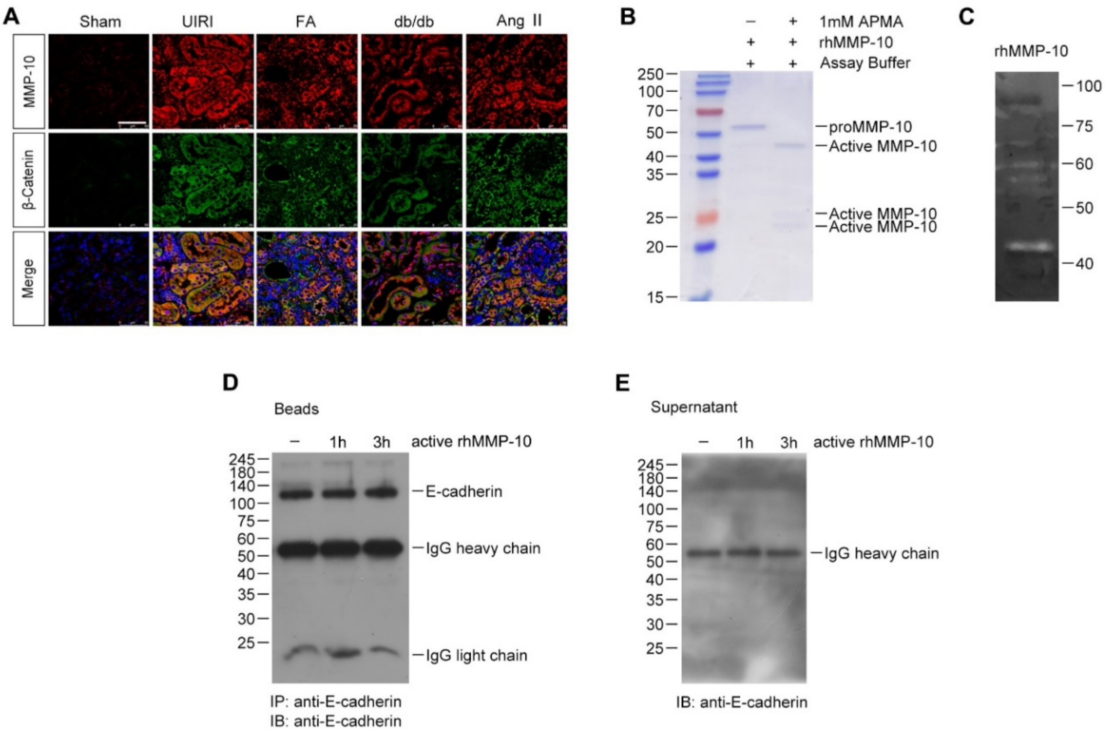

Supplementary Figure S2. E-cadherin is not a direct substrate of MMP-10. (**A**) In various experimental animal models, representative images of double immunofluorescence staining for MMP-10 (red) and β-catenin (Green) were shown. Scar bar, 50 μm. (**B**) The recombinant human proMMP-10 protein was activated in a buffer solution containing 1 mM APMA. (**C**) The enzymatic activity of the rhMMP-10 protein after activation was demonstrated through gelatin zymography. (**D**) Western blot analysis revealed that active MMP-10 did not degrade E-cadherin protein precipitated by anti-E-cadherin antibody. The overall protein level of E-cadherin after incubation with active MMP-10 remained unchanged. (**E**) Western blot analysis revealed no enzymatic hydrolysis of E-cadherin protein by active MMP-10. No notable fragments of E-cadherin were detected in the supernatants.

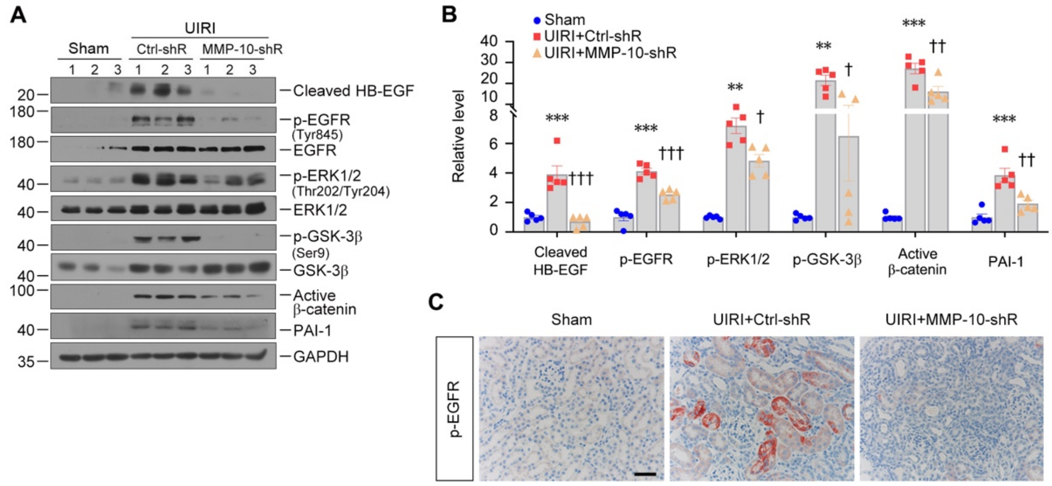

Supplementary Figure S3. Knockdown of MMP-10 inhibits EGFR-mediated transactivation of β-catenin in unilateral ischemia-reperfusion injury (UIRI). (**A**, **B**) Representative Western blots and graphic presentations showed the renal expression of cleaved HB-EGF, p-EGFR (Tyr845), total EGFR, p-ERK1/2 (Tyr202/Tyr204), total ERK1/2, p-GSK3β (Ser9), total GSK3β, active β-catenin, and PAI-1 in different groups as indicated. Data are presented as the mean ± SEM. $^{**}P < 0.01$, $^{***}P < 0.001$ versus sham controls (n = 5). $^{\dagger}P < 0.05$, $^{\dagger\dagger}P < 0.01$, $^{\dagger\dagger\dagger}P < 0.001$ versus UIRI injected with Ctrl-shRNA (n = 5). (**C**) Immunohistochemical staining showed tubular phosphorylated EGFR in various groups as indicated. Scale bar, 50 µm.

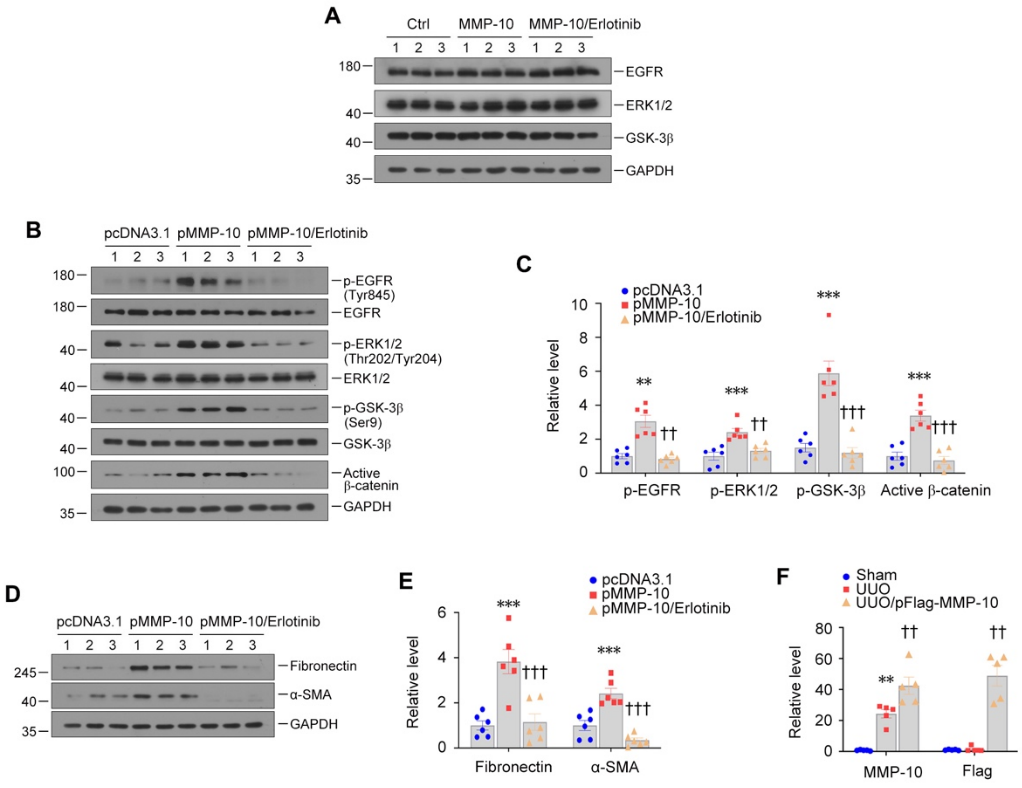

Supplementary Figure S4. Blockade of EGFR activation by erlotinib abolishes MMP-10-induced β-catenin activation. (**A**) Representative Western Blots analyses illustrated that neither rhMMP-10 nor erlotinib affected the protein levels of total EGFR, ERK1/2, and GSK-3β. (**B**, **C**) Erlotinib inhibited β-catenin activation in HK-2 cells after transfection of MMP-10 expression vector. Representative Western blots and graphic presentations showed protein expression of p-EGFR (Tyr845), EGFR, p-ERK1/2 (Thr202/Tyr204), ERK1/2, p-GSK-3β (Ser9), GSK-3β, and active β-catenin after various treatments in HK-2 cells. Data are presented as the mean ± SEM. $^{**}P < 0.01$, $^{***}P < 0.001$ versus controls (n = 6). $^{††}P < 0.01$, $^{†††}P < 0.001$ versus the group with pFlag-MMP-10 transfection alone (n = 6). (**D**, **E**) Representative Western blots and graphic presentations showed fibronectin and α-SMA levels in different groups as indicated. Data are presented as the mean ± SEM. $^{***}P < 0.001$ versus controls (n = 6). $^{†††}P < 0.001$ versus the group with pFlag-MMP-10 transfection alone (n = 6). (**F**) Quantitative data of MMP-10 and Flag in UUO mice after plasmid injection. Data are presented as the mean ± SEM. $^{**}P < 0.01$ versus sham controls (n = 5). $^{††}P < 0.01$ versus UUO plus pcDNA3.1 group (n = 5).

Supplementary Table S1. The sources of antibodies used in this study

Antibodies	Catalogue number	Company	Location
**Primary antibodies**			
Anti-MMP-10	AF-910 (For IHC/IF)	R&D SYSTEMS	Minneapolis, MN
Anti-MMP-10	sc-80197 (For WB)	Santa Cruz Biotechnology	Santa Cruz, CA
Anti-flag	F1804	Sigma-Aldrich	St. Louis, MO
Anti-LTL	FL-1321	VECTOR Laboratories	San Francisco, CA
Anti-PNA	FL-1071	VECTOR Laboratories	San Francisco, CA
Anti-DBA	FL-1031	VECTOR Laboratories	San Francisco, CA
DAPI	C1006	Beyotime	Shanghai, China
Anti-fibronectin	F3648	Sigma-Aldrich	St. Louis, MO
Anti-α-SMA	A2547	Sigma-Aldrich	St. Louis, MO
Anti-vimentin	Ab8978	Abcam	Cambridge, MA
Anti-collagen I	Ba0325	Boster Biotechnology	Wuhan, China
Anti-KIM-1	BA3537 (For WB)	Boster Biotechnology	Wuhan, China
Anti-KIM-1	AF1817 (For IHC)	R&D SYSTEMS	Minneapolis, MN
Anti-active β-catenin	#4270	Cell Signaling Technology	Danvers, MA
Anti-β-catenin	610154	BD biosciences	San Jose, CA
Anti-PAI-1	AF1786	R&D SYSTEMS	Minneapolis, MN
Anti-MMP-7	GTX104658	GeneTex	Irvine, CA
Anti-GAPDH	RM2002	Ray Antibody Biotech	Beijing, China
Anti-α-tubulin	RM2007	Ray Antibody Biotech	Beijing, China
Anti-β-actin	RM2001	Ray Antibody Biotech	Beijing, China
Anti-E-cadherin	Ab76055 (For WB)	Abcam	Cambridge, MA
Anti-E-cadherin	14472S (For IP)	Cell Signaling Technology	Danvers, MA
Anti-HB-EGF	sc-74526	Santa Cruz Biotechnology	Santa Cruz, CA
Anti-p-EGFR (Tyr845)	#2231 (For IHC)	Cell Signaling Technology	Danvers, MA
Anti-p-EGFR (Tyr845)	sc-57542 (For WB)	Santa Cruz Biotechnology	Santa Cruz, CA
Anti-EGFR	sc-373746	Santa Cruz Biotechnology	Santa Cruz, CA
Anti-p-GSK-3β (Ser9)	#9336	Cell Signaling Technology	Danvers, MA
Anti-GSK-3β	#9315	Cell Signaling Technology	Danvers, MA
Anti-ERK1/2	#4695	Cell Signaling Technology	Danvers, MA
Anti-p-ERK1/2 (Tyr202/Tyr204)	#9101	Cell Signaling Technology	Danvers, MA
**Secondary antibodies**			
Goat anti-mouse	BA1050 (For WB)	Boster Biotechnology	Wuhan, China
Goat anti-rabbit	BA1054 (For WB)	Boster Biotechnology	Wuhan, China
Rabbit anti-goat	BA1060 (For WB)	Boster Biotechnology	Wuhan, China

Supplementary Table S1 (Continued). The sources of antibodies used in this study

Antibodies	Catalogue number	Company	Location
Donkey Anti-Mouse	715-0165-150 (For IHC)	Jackson ImmunoResearch	West Grove, PA
Donkey Anti-Rabbit	711-065-152 (For IHC)	Jackson ImmunoResearch	West Grove, PA
Donkey Anti-Goat	705-065-147 (For IHC)	Jackson ImmunoResearch	West Grove, PA
Donkey Anti-Mouse	715-225-150 (For IF)	Jackson ImmunoResearch	West Grove, PA
Donkey Anti-Rabbit	711-1165-152 (For IF)	Jackson ImmunoResearch	West Grove, PA
Donkey Anti-Goat	705-165-003 (For IF)	Jackson ImmunoResearch	West Grove, PA

Supplementary Table S2. The nucleotide sequences of primers used for qRT-PCR in this study

Gene	Species	Primer Sequence 5' to 3'	
		Forward	Reverse
Mmp10	Mouse	GACCCCACTCACTTTCTCCA	GGAATAAGTTGGTCCCTGAGG
MMP10	Human	CCACTCTACAACTCATTCACA	TGAATGCCATTCACATCATCTTG
WNT1	Human	ACCCAATCCCTCTCCACTCT	GATTCAAGGAAAAGCCACCA
WNT2	Human	GTGGATGCAAAGGAAAGGAA	AGCCAGCATGTCCTGAGAGT
WNT2B	Human	GTGTCCTGGCTGGTTCCTTA	AGCTGGTGCAAAGGAAAGAA
WNT3	Human	TGTGAGGTGAAGACCTGCTG	AAAGTTGGGGGAGTTCTCGT
WNT3A	Human	CAAGATTGGCATCCAGGAGT	ATGAGCGTGTCACTGCAAAG
WNT4	Human	ATGGAAGTCACACCCTCTGG	CCTGGAAGGACCCACAGATA
WNT5A	Human	GGGTGGGAACCAAGAAAAAT	TGGAACCTACCCATCCCATA
WNT5B	Human	AAGAAGTGCACGGAGATCGT	CACCCACCAAGAGGAGAGAA
WNT6	Human	GTCACGCAGGCCTGTTCTAT	CGTCCATAAAGAGCCTCGAC
WNT7A	Human	CCCACCTTCCTGAAGATCAA	ACAGCACATGAGGTCACAGC
WNT7B	Human	TCAACGAGTGCCAGTACCAG	CCCTCGGCTTGGTTGTAGTA
WNT8A	Human	GAACTGCCCTGAAAATGCTC	ATCCTTTCCCCAAATTCCAC
WNT9A	Human	GCAAGCATCTGAAGCACAAG	TGCTCTCGCAGTTCTTCTCA
WNT9B	Human	GAGGACTCACCCAGCTTCTG	TAGGCCTAGTGCTTGCAGGT
WNT10A	Human	GGTTGCTCCACACCCTAAAA	ATGATGAAGGGAATGGTGGA
WNT10B	Human	TCTGACAAGGGGACAGAACC	TCATTGCTTAGAGCCCGACT
WNT11	Human	CAGGCAGTGCAACAAGACAT	TGAGGGTCCTTGAGCAGAGT
WNT16	Human	AAATGCGCAGGAGAGAAAAA	ACCCTCTGATGTACGGTTGC
ACTB	Human	CTCACCATGGATGATGATATCGC	AGGAATCCTTCTGACCCATGC
Actb	Mouse	CAGCTGAGAGGGAAATCGTG	CGTTGCCAATAGTGATGACC
