# Supplementary material for: Matrix metalloproteinase-10 promotes kidney fibrosis by transactivating β-catenin signaling
Source: Cell Death Discov. 2025 May 17;11:241. doi: 10.1038/s41420-025-02521-w (PMC12085647; doi:10.1038/s41420-025-02521-w)

Original Western Blot

Figure 1B

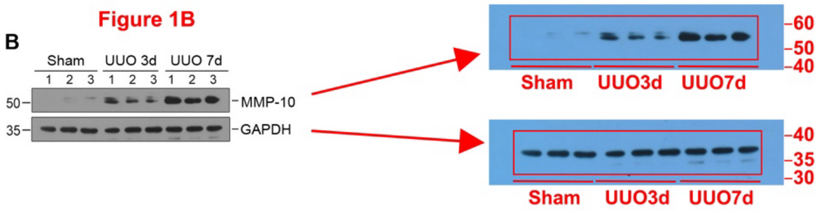

Figure 1F

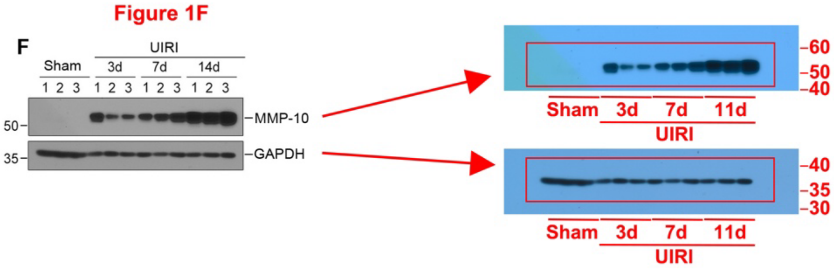

Figure 1J

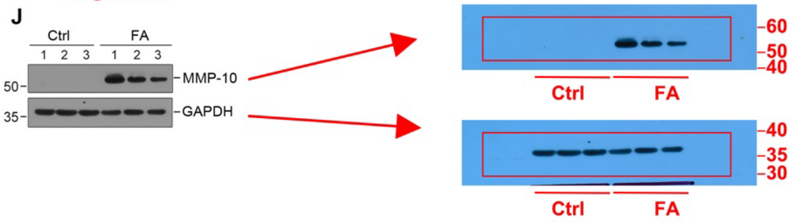

Original Western Blot

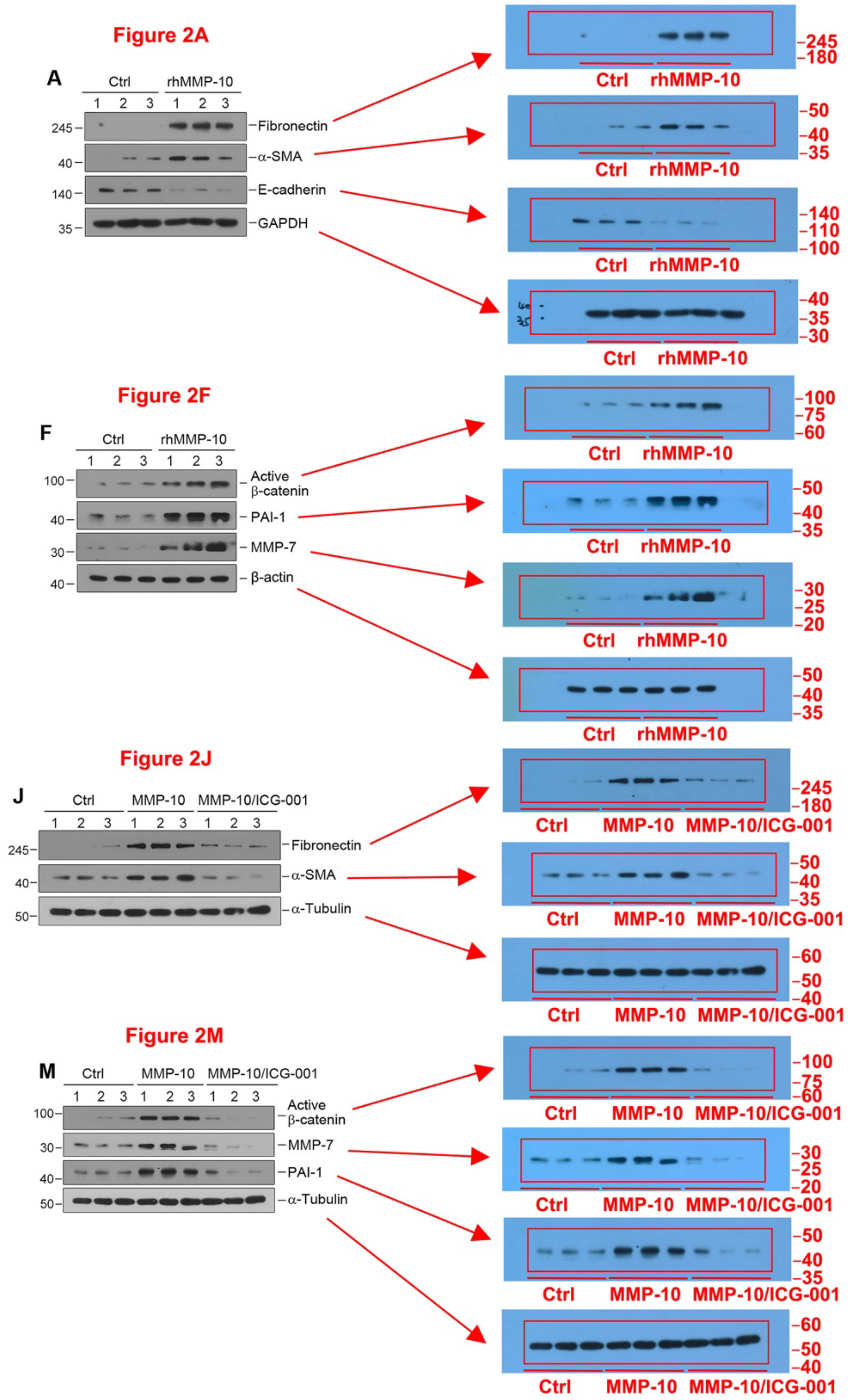

Original Western Blot

Figure 3D

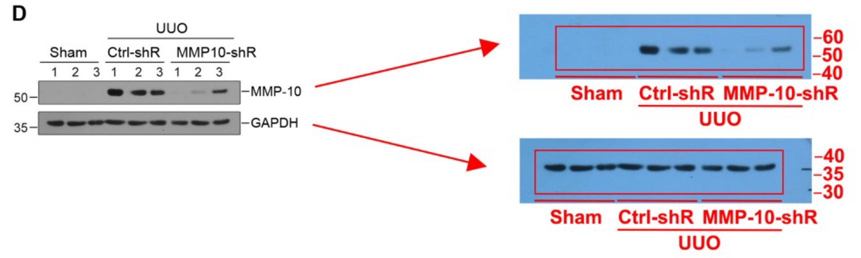

Figure 3F

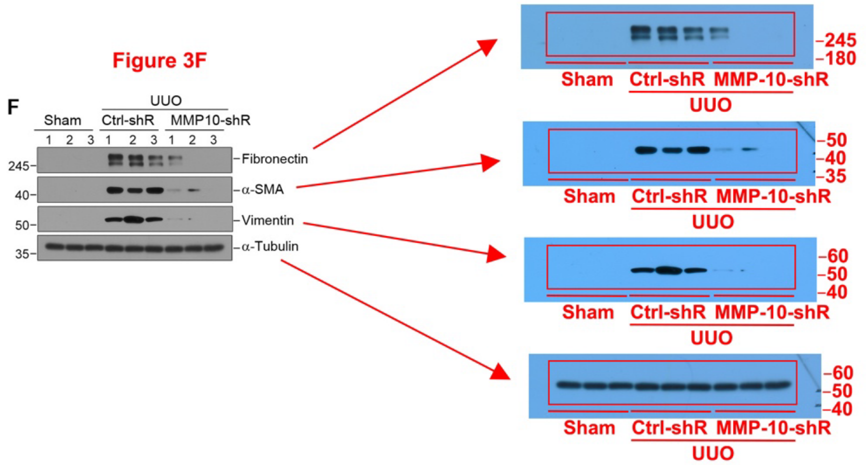

Figure 3H

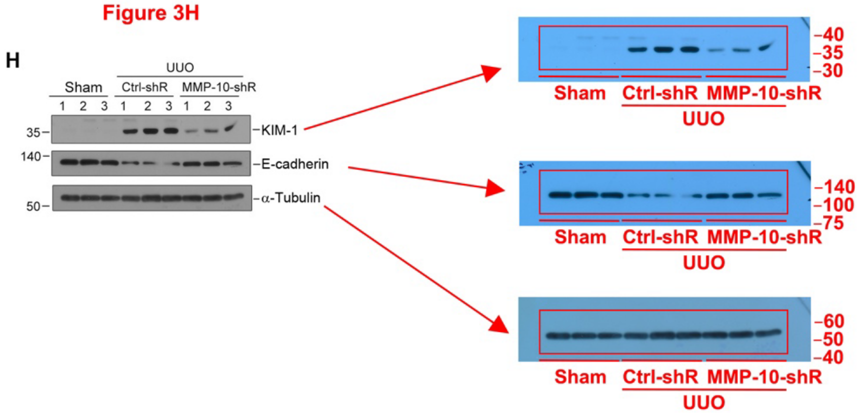

Original Western Blot

Figure 4B

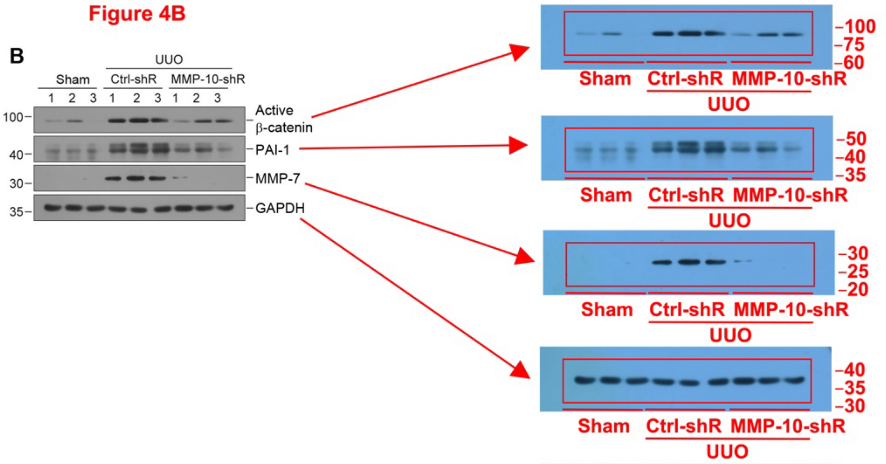

Figure 4H

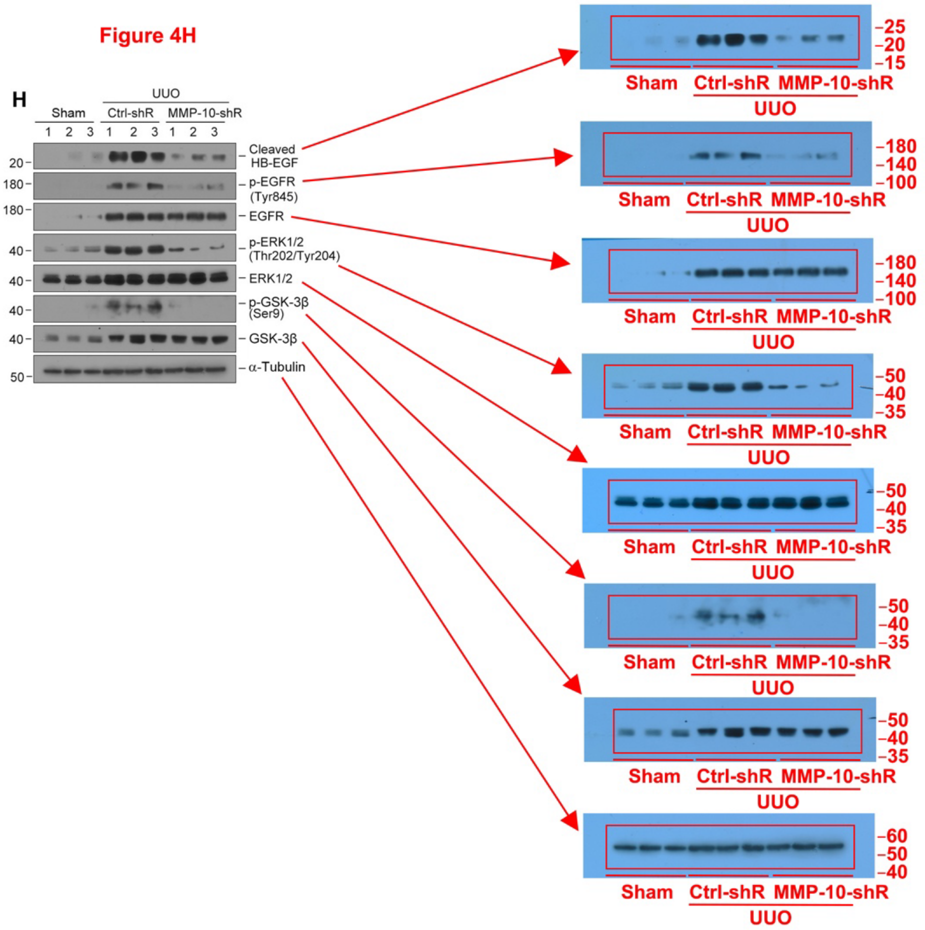

Original Western Blot

Figure 5B

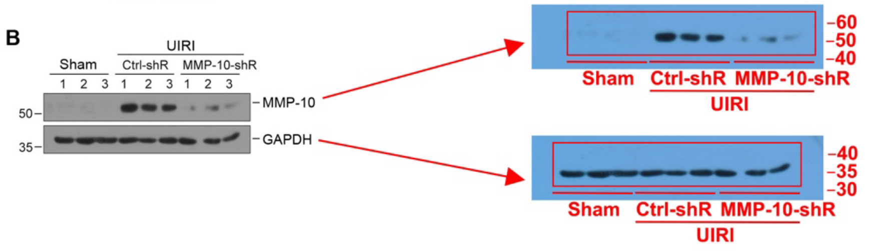

Figure 5G

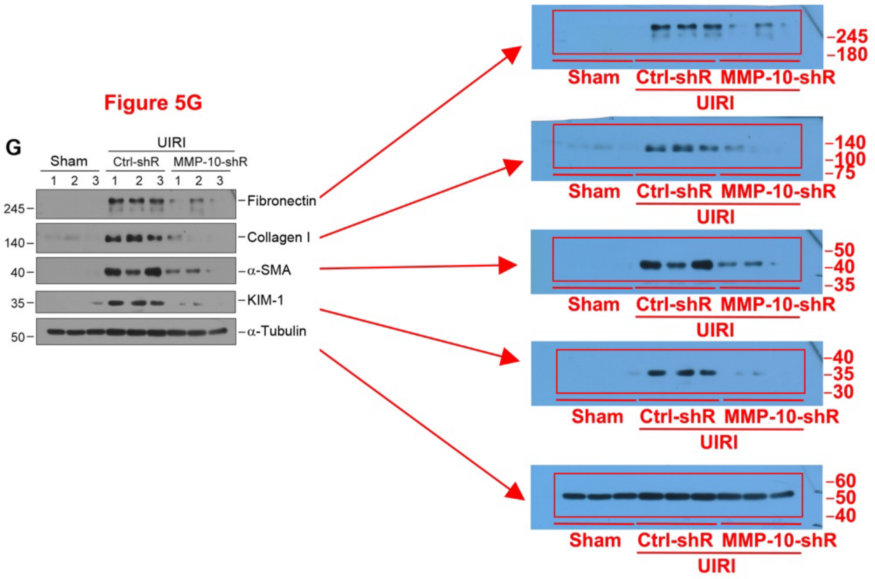

Original Western Blot

Figure 6A

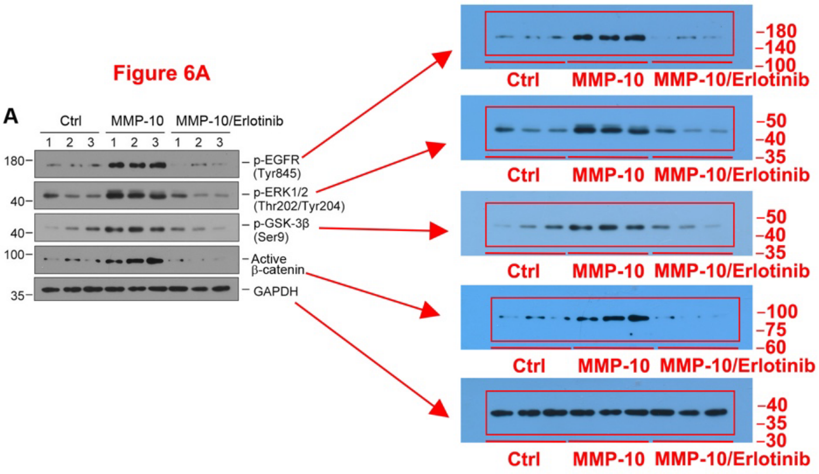

Figure 6D

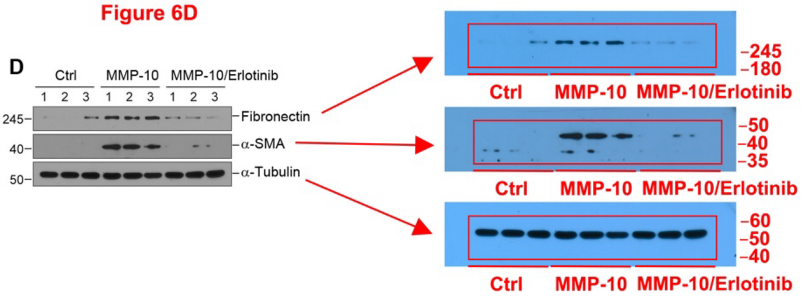

Figure 6G

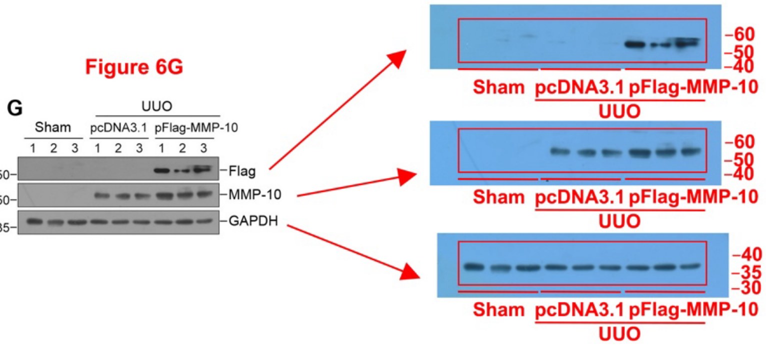

Original Western Blot

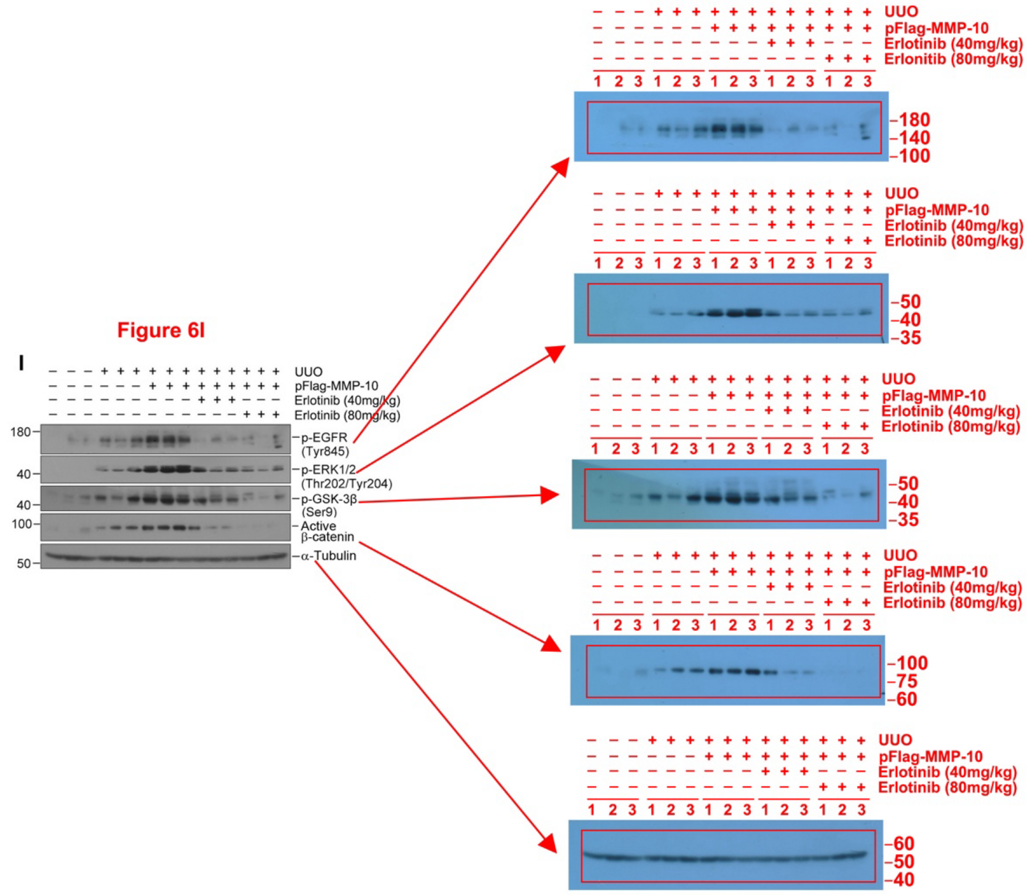

Original Western Blot

Figure 7B

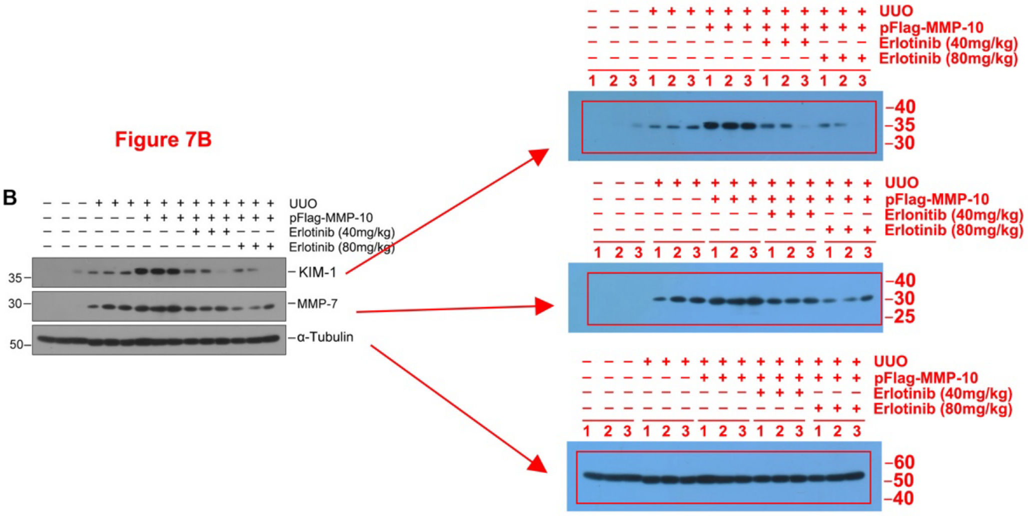

Figure 7D

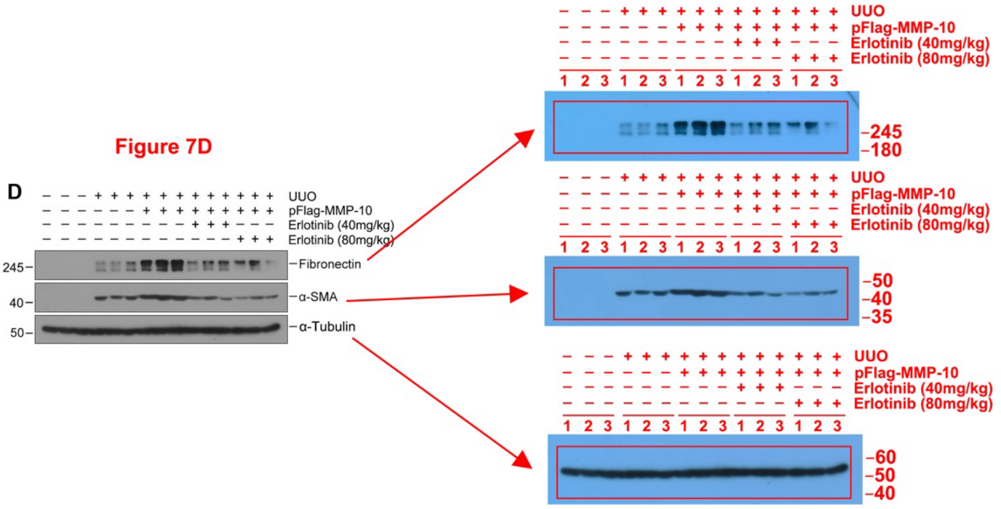

Original Western Blot

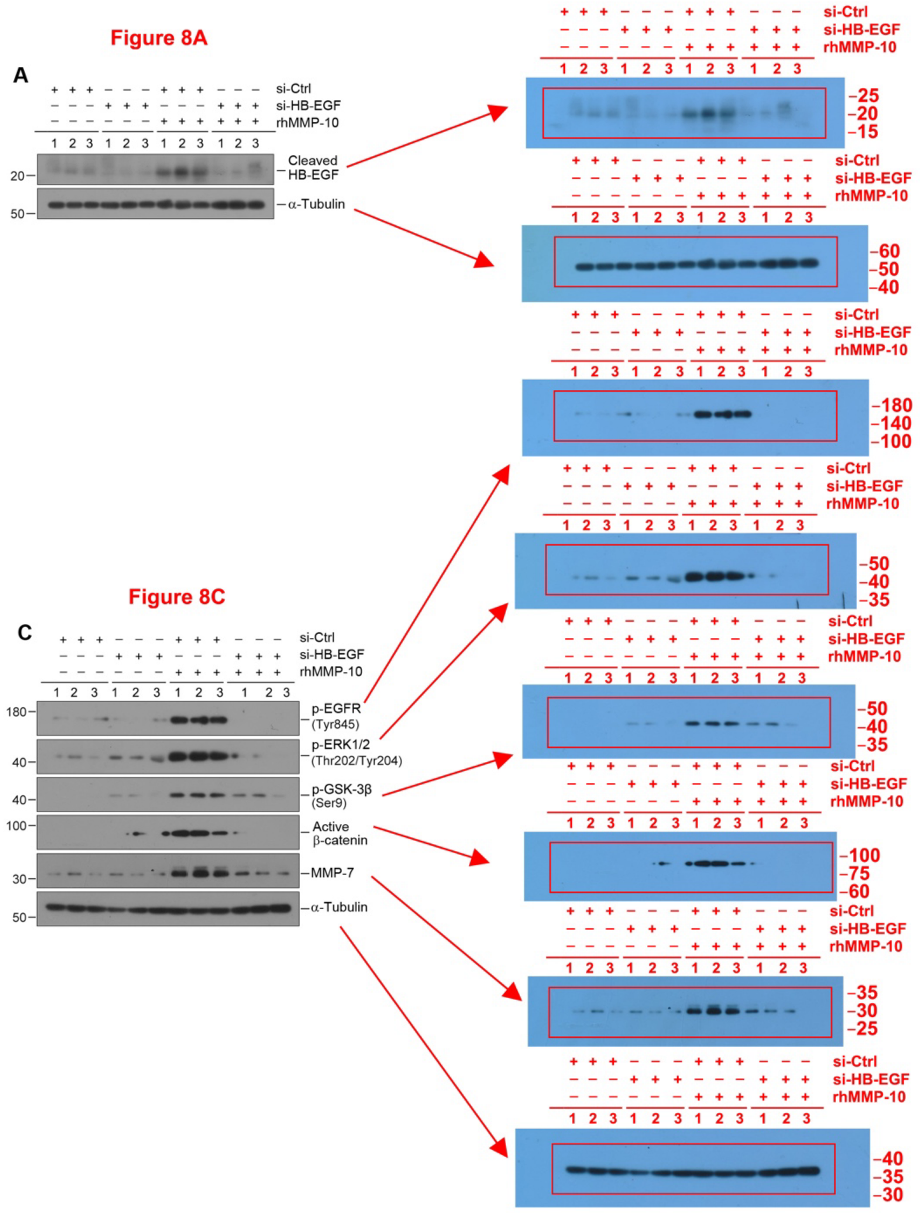

Supplemental Western Blot Original file

Figure 8F

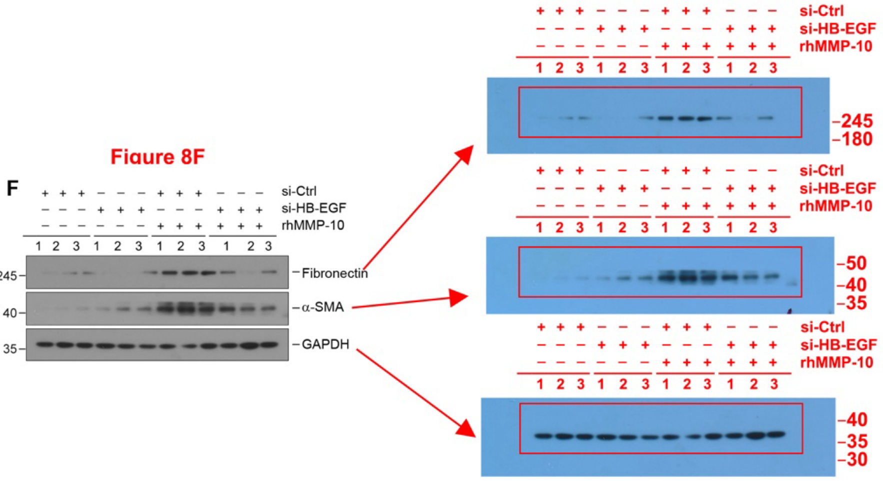

Figure 8I

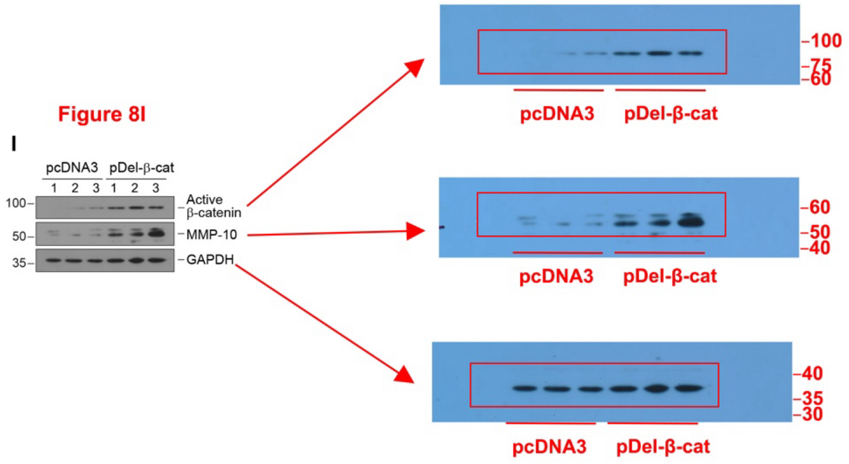

Supplemental Western Blot Original file

Supplementary Figure S2D

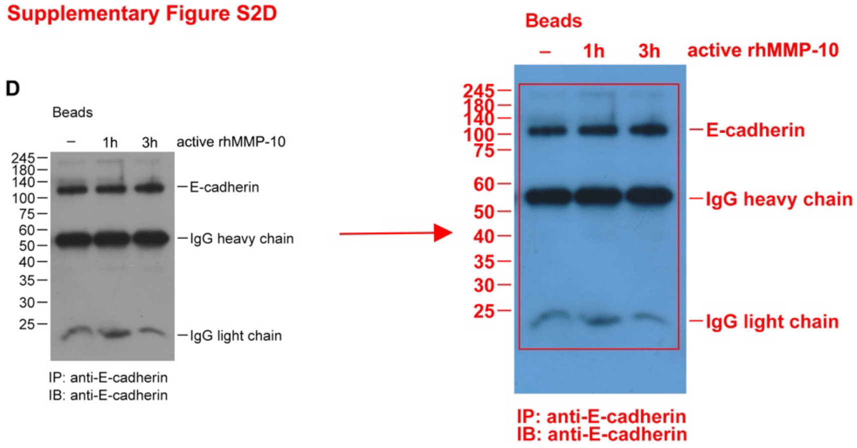

Supplementary Figure S2E

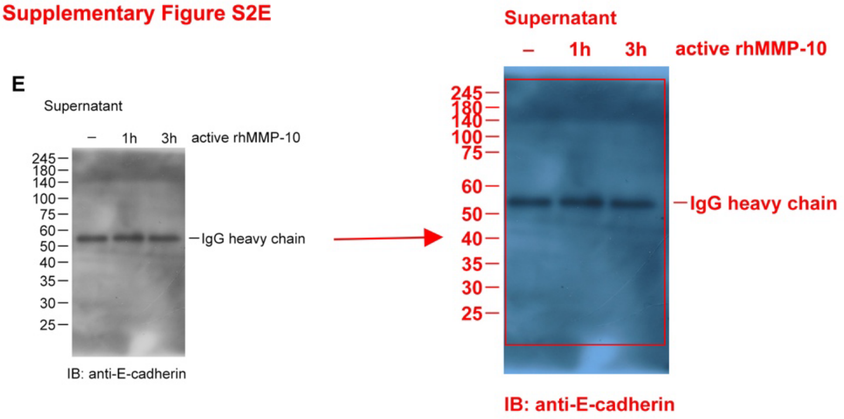

Supplemental Western Blot Original file

Supplementary FIgure S3A

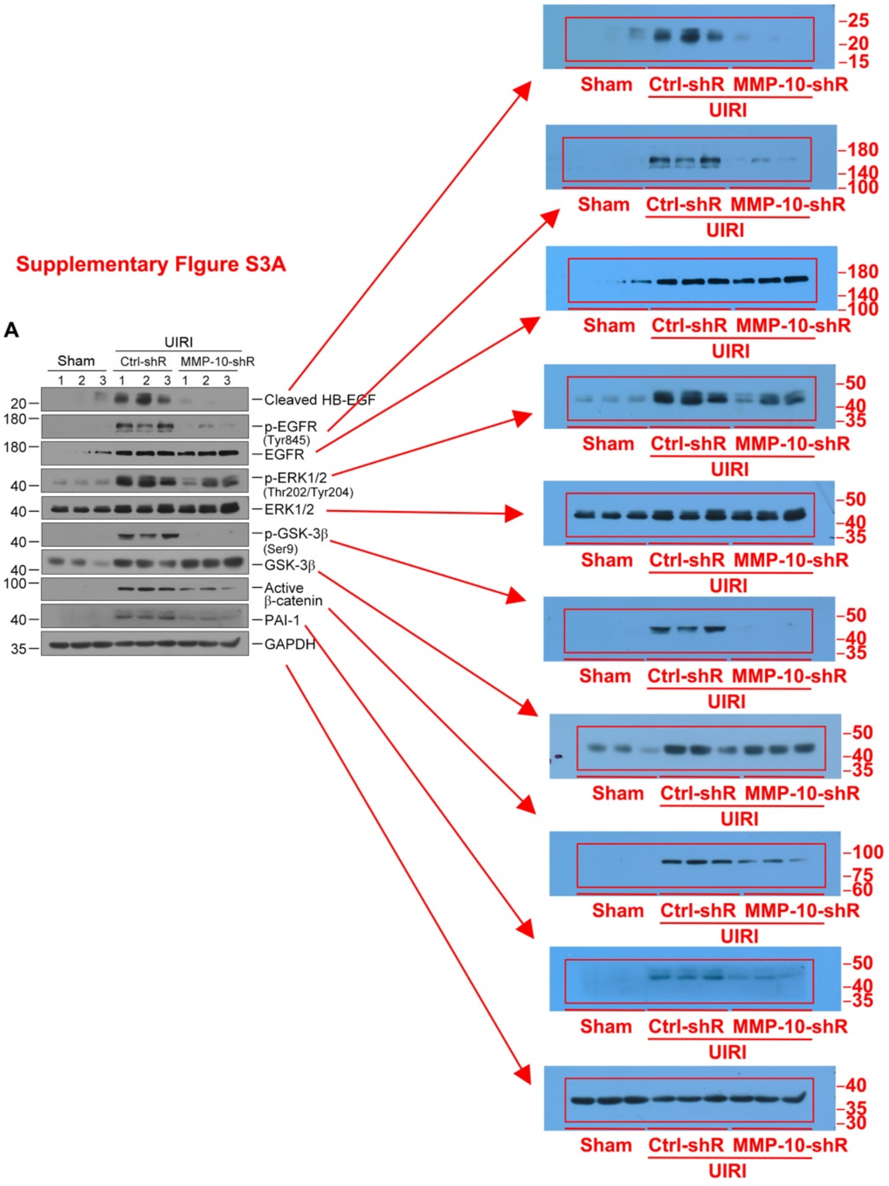

Supplemental Western Blot Original file

Supplementary Figure S4A

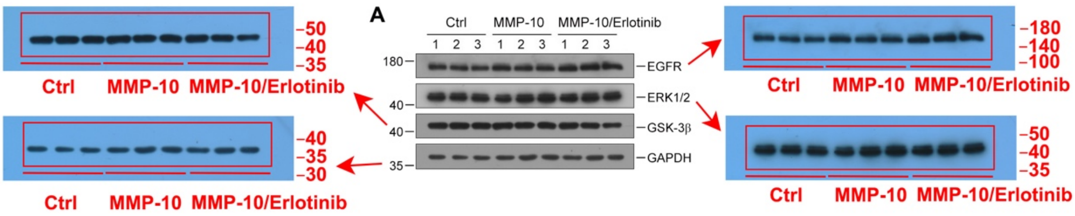

Supplementary Figure S4B

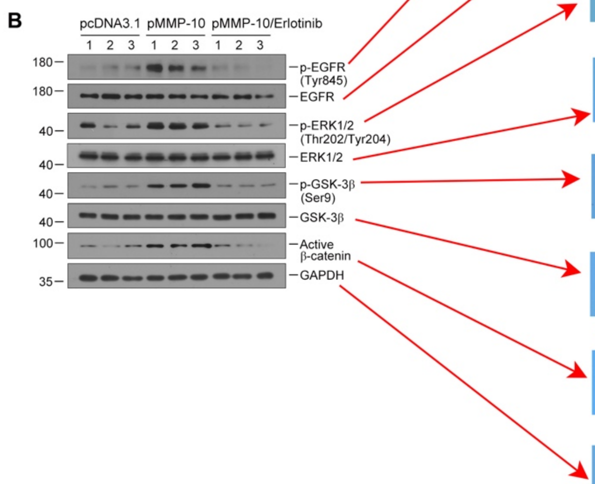

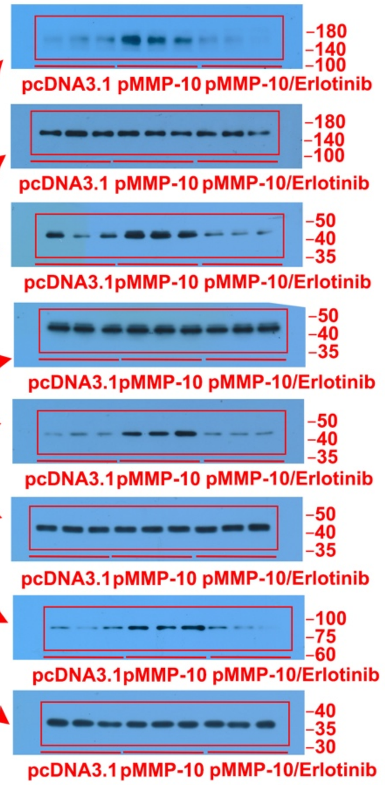

Supplementary Figure S4D

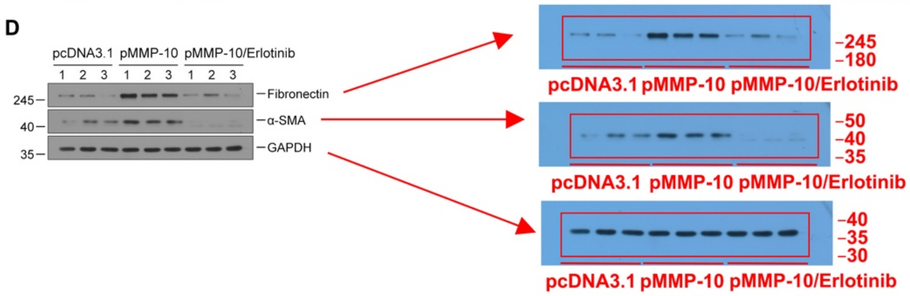

Supplement: Supplementary file 2 — Original Western blots [file 41420_2025_2521_MOESM2_ESM.pdf]
